# Alkaline phosphatase LapA regulates quorum sensing–mediated virulence and biofilm formation in *Pseudomonas aeruginosa* PAO1 under phosphate depletion stress

**DOI:** 10.1128/spectrum.02060-23

**Published:** 2023-10-05

**Authors:** Xiaojuan Tan, Xi Cheng, Jingjing Xiao, Qianqian Liu, Dongsheng Du, Minghui Li, Yang Sun, Jinwei Zhou, Guoping Zhu

**Affiliations:** 1 Anhui Provincial Key Laboratory of Molecular Enzymology and Mechanism of Major Diseases, College of Life Sciences, Anhui Normal University, Wuhu, Anhui, China; 2 School of Food and Biology Engineering, Xuzhou University of Technology, Xuzhou, Jiangsu, China; Yangzhou University, Yangzhou, Jiangsu, China

**Keywords:** *Pseudomonas aeruginosa*, alkaline phosphatase LapA, virulence, biofilms, chronic wound infection

## Abstract

Among various environmental stresses, the depletion of inorganic phosphate, which is an important signaling molecule that modulates virulence production, is notable. In our previous study, we demonstrated that *lap*A, encoding alkaline phosphatase, plays a key role in *Pseudomonas aeruginosa* biofilm formation in porcine skin explants, an *ex vivo* chronic wound model. However, the mechanism by which *lap*A participates in the virulence and biofilm formation of *P. aeruginosa* remains unclear. In the present study, the *lap*A deletion mutant was constructed in *P. aeruginosa* PAO1, and the phenotypic characterization was assessed. Our data demonstrated that phosphate depletion stress significantly enhanced elastase activity, hemolysis, rhamnolipid production, swarming and swimming motilities, and 3-oxo-C12-homoserine lactone (HSL) production in *P. aeruginosa*. Moreover, phosphate starvation evidently induced the virulence of *P. aeruginosa* in *Caenorhabditis elegans* (*C. elegans*) through fast-kill and slow-kill infections. Deletion of the *lap*A gene led to broad phenotypic changes, including reduced elastase activity, swimming motility, C4-HSL, and 3-oxo-C12-HSL production, as well as increased chitinase activity and rhamnolipid production under phosphate depletion stress, which was unrelated to C4-HSL. Deletion of the *lap*A gene also significantly inhibited PAO1 biofilm formation in porcine skin explants by reducing the expression levels of *las* and *rhl* quorum sensing systems and extracellular polymeric substance synthesis. Finally, *lap*A deletion also reduced the virulence of PAO1 in *C. elegans* in fast-kill and slow-kill infection assays. Thus, the findings suggest that targeting *lap*A can help control biofilm formation and reduce virulence in nonhealing-infected wounds, where *P. aeruginosa* is a persistent problem.

**IMPORTANCE:**

Our previous study demonstrated that the expression of lapA was induced under phosphate depletion conditions, but its roles in virulence and biofilm formation by *Pseudomonas aeruginosa* remain largely unknown. This study presents a systematic investigation of the roles of lapA in virulence induction and biofilm formation by constructing a lapA-deficient strain with *P. aeruginosa* PAO1. The results showed that deletion of the lapA gene evidently reduced elastase activity, swimming motility, C4-HSL, and 3-oxo-C12-HSL production, and increased rhamnolipid production under phosphate depletion stress. Moreover, lapA gene deletion inhibited PAO1 biofilm formation in porcine skin explants by reducing the expression levels of las and rhl quorum sensing systems and extracellular polymeric substance synthesis. Finally, lapA gene deletion also reduced the virulence of PAO1 in *Caenorhabditis elegans* in fast-kill and slow-kill infection assays. This study provides insights into the roles of lapA in modulating *P. aeruginosa* virulence and biofilm formation under phosphate depletion stress.

## INTRODUCTION

*Pseudomonas aeruginosa* is a ubiquitous opportunistic bacterial pathogen that causes infection in a wide category of patients, such as those with burns, surgical wounds, lung diseases, or immunocompromised diseases (1). *P. aeruginosa* has been a significant pathogen owing to its intrinsic ability to develop antibiotic resistance, form biofilms, and release a high amount of virulence factors (2). The pathogenicity of *P. aeruginosa* is commonly attributed to its different types of virulence factors, mainly including phenazines, elastase, hemolysis, rhamnolipid, lipopolysaccharides, motilities, and secretion systems, which play important roles in the development of *P. aeruginosa* infections (3 – 6). Biofilms developed by *P. aeruginosa* cause many persistent and noninvasive human infections, such as chronic wound infection, cystic fibrosis, and medical device–associated infections (7, 8).

Quorum sensing (QS) in bacteria is a key player in regulating the expression of virulence genes and biofilm formation (9, 10). *P. aeruginosa* contains at least three known QS systems, namely, *las*, *rhl*, and *pqs*, that control the expression of more than 300 genes involved in virulence factor production and biofilm formation (11, 12). *las* and *rhl* are N-acylated homoserine lactone (AHL) signaling systems. In the *las* system, N-(3-oxo-dodecanoyl)-L-homoserine lactone (3-oxo-C12-HSL) produced by autoinducer synthase LasI interacts with the LasR receptor to activate certain virulence factors, including LasB elastase, LasA protease, exotoxin A, and alkaline protease (13). Similarly, in the *rhl* system, N-butanoyl-L-homoserine lactone (C4-HSL) produced by autoinducer synthase RhlI interacts with RhlR to induce other virulence factors such as rhamnolipid, pyocyanin, chitinase, and hydrogen cyanide (11, 13, 14). 2-Heptyl-3-hydroxy-4-quinolone is the third primary QS signal, as well as two AHLs, in *P. aeruginosa* that functions as a signaling molecule by binding to its cognate receptor PqsR to induce pyocyanin production (13, 14).

Environmental stresses such as osmotic stress, starvation, and iron and phosphate depletion can promote the production of virulence factors thus allowing *P. aeruginosa* to cause acute or chronic infections (5, 15). Bazire et al. reported that phosphate limitation reduced 3-oxo-C12-HSL and C4-HSL concentrations while allowing rhamnolipid hyper-production (16). However, Lee et al. demonstrated that phosphate limitation could activate the *rhl* and *pqs* QS systems in the absence of a functional *las* system (4, 17). Soto-Aceves et al. demonstrated that the *rhl* system is at the predominant regulating hierarchy in *P. aeruginosa* PAO1 under phosphate-limiting conditions (18). Based on previous studies, the effects of phosphate depletion on *las* and *rhl* systems in *P. aeruginosa* remain not fully understood.

Phosphate is an essential element for all living cells; it is a vital component of the energy molecule ATP, nucleic acid, membrane phospholipids, and other biomolecules. In addition to its metabolic importance, inorganic phosphate is an important signaling molecule that modulates virulence in different pathogens (4, 19, 20). Substantial depletion of phosphate can occur after surgical injury, which evidently increases the virulence of *P. aeruginosa* (5, 21, 22). Our previous study demonstrated that the expression of *lap*A, encoding alkaline phosphatase, was highly induced when *P. aeruginosa* biofilm developed in porcine skin explants, a chronic skin wound model (22). Meanwhile, alkaline phosphatase activity was highest in mature biofilms formed in the *ex vivo* wound model (22). Therefore, our data suggested that *lap*A would play an important role in the biofilm formation of *P. aeruginosa* in chronic wounds. Ball et al. demonstrated that *lap*A expression was induced when *P. aeruginosa* was incubated under phosphate depletion conditions (23, 24). As shown in the previous studies, porcine skin explants provide a phosphate-depleted environment to investigate wound infection caused by *P. aeruginosa*. However, the mechanism by which *lap*A participates in virulence induction and biofilm formation of *P. aeruginosa* remains unclear. Therefore, in the present study, as an extension of our previous discovery, a mutant with *lap*A gene deletion was constructed in *P. aeruginosa* PAO1 to further study the role of this gene in virulence factor production and biofilm formation by *P. aeruginosa*. Phenotypic characterization of the *lap*A-deficient strain showed significant roles of elastase activity, swimming motility, C4-HSL and 3-oxo-C12-HSL production, rhamnolipid production, and biofilm formation. These results present novel insights into how LapA modulates *P. aeruginosa* virulence factor production and biofilm formation under phosphate starvation conditions.

## RESULTS

### *Pseudomonas aeruginosa* virulence factors are activated at phosphate-depleted stress

To understand the effects of phosphate depletion on *P. aeruginosa* virulence, elastase and chitinase activity, hemolysis, and rhamnolipid production were measured in *P. aeruginosa* PAO1 grown under phosphate-depleted [protease-peptone (PP) medium] and phosphate-rich [lysogeny broth (LB) medium] conditions. Elastase activity, hemolysis, and rhamnolipid production, all of which are controlled by the *las* and *rhl* systems (12), were significantly enhanced when grown under phosphate-depleted conditions compared with those of phosphate-rich conditions (Fig. S1A through C). Different from the results of elastase activity, hemolysis, and rhamnolipid production, chitinase activity was downregulated under phosphate-depleted stress (Fig. S1D). However, very less pyocyanin was produced in the PP medium (Fig. S1E), mainly because the synthesis of pyocyanin is affected by the carbon and nitrogen sources present in the fermentation medium, in addition to QS systems and the presence or absence of phosphate (25).

Bacterial motility is another QS-mediated virulence phenotype that involves pili, flagella, and rhamnolipid to enhance *P. aeruginosa* infection (26, 27). We next analyzed the effect of phosphate on the swarming and swimming motilities of PAO1. After 15-h culture in the swarming medium and 24-h culture in the swimming medium, the results showed that phosphate depletion resulted in an evident increase in swarming and swimming (average increase to 1.8 times), while no swarming phenotype was found under phosphate-rich conditions (Fig. S2).

To determine the effects of phosphate-depleted stress on *las* and *rhl* systems, we compared the levels of C4-HSL and 3-oxo-C12-HSL produced by PAO1 grown in the PP medium and LB medium through high-performance liquid chromatography (HPLC) assays. As shown in Fig. S3, C4-HSL levels produced by PAO1 in the PP medium were reduced to 57.8% compared with those in the LB medium, whereas 3-oxo-C12-HSL levels produced in the PP medium showed a slight increase. Moreover, the results from AHL reporter plate bioassay indicated that phosphate-depleted stress reduced C4-HSL production when compared with phosphate-rich conditions (data are shown below), a finding consistent with the results of HPLC assays. Therefore, these results from virulence phenotypic screens indicate that phosphate depletion plays positive roles in regulating elastase activity, hemolysis, rhamnolipid production, and motility, while its roles in C4-HSL production and chitinase activity are negative. Surprisingly, lesser levels of C4-HSL did not result in lesser virulence of *P. aeruginosa* PAO1 under phosphate depletion conditions.

### Phosphate-depleted stress increases the virulence of *P. aeruginosa* in animal infection models

Our finding that *P. aeruginosa* virulence was increased under phosphate depletion conditions indicated that phosphate-depleted stress could occur under non-laboratory conditions, such as during host infection. To probe this possibility, we assessed the relative pathogenicity of the PAO1 strain in *Caenorhabditis elegans* fast-kill and slow-kill (SK) infection assays under phosphate-depleted and phosphate-rich conditions, respectively.

Fast-killing is a toxin-mediated mode of death that depends on a diffusible toxin produced by *P. aeruginosa* (28). PGS agar (1% peptone, 1% glucose, 1% NaCl, 150 mM sorbitol, and 1.7% agar), which is a high-osmotic stress and low-phosphate medium, was used as a medium for fast-kill infection assays. In *C. elegans* fast-kill infection assays, the survival rate of worms was only 20% in the PGS medium, whereas the survival rate was increased to 60% in the phosphate-rich medium (PGS + Pi) when fed on PAO1 for 30 h (Fig. S4A). Moreover, the number of visible bacteria in the plates containing the PGS medium was reduced by 2 log compared with that in plates containing the PGS + Pi medium (Fig. S4B). Meanwhile, the color of the plates containing PAO1 with PGS was deep purple, whereas the color of the plates containing PAO1 with PGS + Pi was yellow, which was the medium color (Fig. S4C); this result was consistent with that of a previous study (29). Therefore, the results indicated that the virulence produced by PAO1 in the PGS medium was attributed to phenazine-1-carboxylic acid instead of pyocyanin.

SK occurs due to an active infection by live *P. aeruginosa* that accumulates in the lumen of the *C. elegans* intestine (28). For SK infection assays, low-osmotic stress and phosphate-rich medium (SK medium) were used, resulting in worm death of several days. In *C. elegans* SK infection assays, the survival rate of worms was only 5% in the SK medium without phosphate (SK-Pi medium) when the worms were fed on PAO1 for 6 days, whereas the survival rate was increased to 40% when the worms were fed on PAO1 in the SK medium under the same conditions (Fig. S5A). In addition, no difference was observed between the number of visible bacteria in the SK and SK-Pi media (Fig. S5B). Interestingly, the color of the plates containing PAO1 in the SK-Pi medium was blue green, whereas the color of the plates containing PAO1 in the SK medium was light green (Fig. S5C). Therefore, the results of SK infection assays revealed that the virulence produced by PAO1 in the SK-Pi medium is attributed to pyocyanin, and phosphate-depleted stress increased pyocyanin production under low osmotic stress. In summary, these results from animal infection assays indicate that phosphate-depleted stress plays a positive role in the pathogenicity of *P. aeruginosa* to *C. elegans*; the virulence factors are different between fast-kill and SK infections.

### LapA is important for alkaline phosphatase production under phosphate depletion conditions

**Fig 1:**
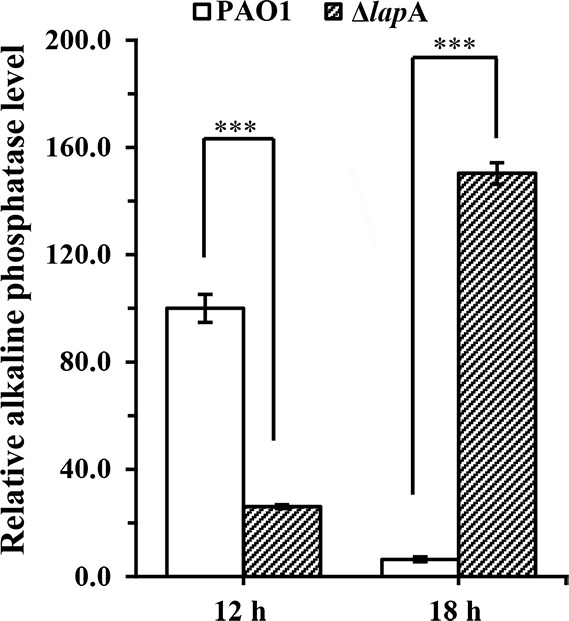
Alkaline phosphatase levels measured in the supernatants from *P. aeruginosa* PAO1 and Δ*lap*A strains under phosphate-depleted conditions. The amount of alkaline phosphatase was defined as micromoles of *p*-nitrophenol liberated from *p*-nitrophenyl phosphate at a specific time point. Data are shown as mean ± standard error of the mean of at least three independent experiments. *P < 0.05, **P < 0.01, ***P < 0.001.

As shown in our previous study, *lap*A is highly expressed in *P. aeruginosa* PAO1 biofilms developed in porcine skin explants, a skin infection model (22). Ball et al. demonstrated that LapA is induced under phosphate-limiting growth conditions (23). However, the authors did not detect alkaline phosphatase secreted by the Δ*lap*A strain during the entire growth phases. In our present study, the Δ*lap*A strain was constructed through the homologous recombination method to investigate the effects of *lap*A on alkaline phosphatase production and virulence of *P. aeruginosa*. Alkaline phosphatase produced by Δ*lap*A and wild-type (WT) strains was measured during the entire growth phases under phosphate-limiting (PP medium) and phosphate-rich (LB medium) conditions. The results showed that the deletion of the *lap*A gene and complementation did not affect PAO1 growth in both media (Fig. S6). When incubating Δ*lap*A and WT strains in the PP medium, no alkaline phosphatase was produced at the early exponential phase (*t* = 4 h). The Δ*lap*A strain had only 26.1% of the total alkaline phosphatase activity secreted by the WT strain at the early stationary phase. Surprisingly, the Δ*lap*A strain continuously produced alkaline phosphatase at the late stationary phase (*t* = 18 h), whereas the WT strain produced very less alkaline phosphatase at 18 h (Fig. 1). Moreover, the introduction of the *lap*A gene on a plasmid to Δ*lap*A strain partially restored the WT morphology under phosphate depletion conditions (Fig. S7). However, neither the Δ*lap*A nor WT strain did produced alkaline phosphatase during the whole growth phases in the LB medium (Fig. S8). We, therefore, conclude that alkaline phosphatase is produced under phosphate-limiting conditions. Meanwhile, we speculate that other gene(s) encoding a nearly identical alkaline phosphatase would be highly expressed at the stationary phase when the *lap*A gene was deficient.

### LapA involvement in the regulation of different virulence phenotypes

To investigate the effects of *lap*A on the virulence of *P. aeruginosa*, elastase and chitinase activity, hemolysis, rhamnolipid production, pyocyanin, and AHLs were subsequently determined in the Δ*lap*A strain in low-phosphate (PP) and high-phosphate (LB) media and then compared with virulence produced by the WT and complementation strains under the same conditions.

**Fig 2:**
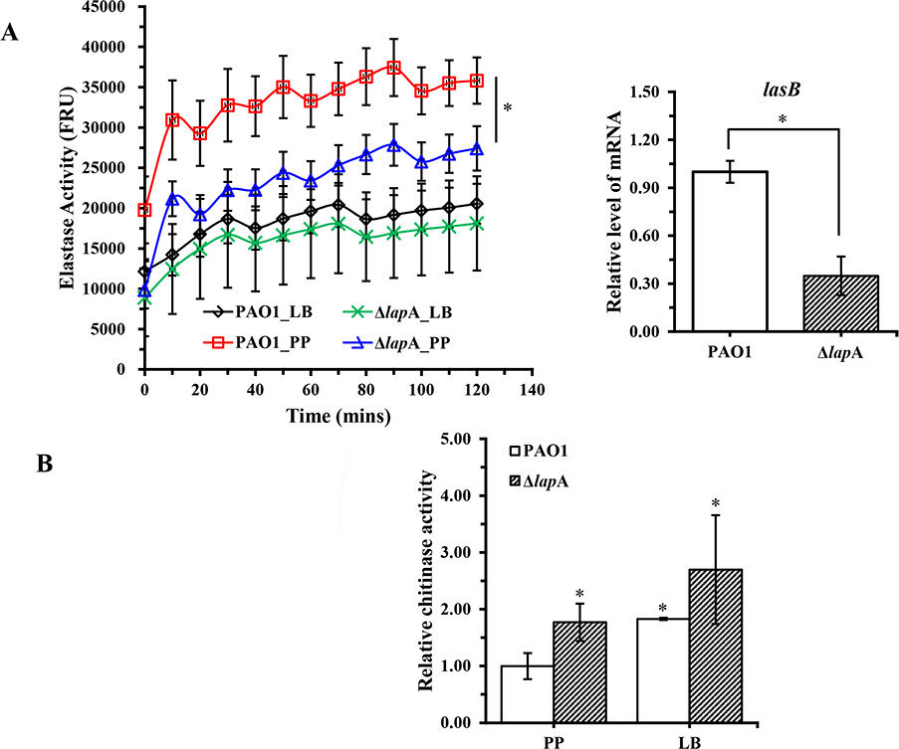
Deletion of the *lap*A gene reduced elastase activity in *P. aeruginosa* PAO1 under phosphate-depleted stress. (**A**) Elastase in the supernatants of WT and Δ*lap*A strains cultured under phosphate-depleted and phosphate-rich conditions for 18 h (left). The relative expression levels of *las*B in WT and Δ*lap*A strains cultured under phosphate-depleted conditions for 18 h were detected by quantitative reverse transcription–polymerase chain reaction assay (right). (**B**) WT and Δ*lap*A strains were cultured in phosphate-depleted and phosphate-rich media for 18 h, and chitinase activity in the pellets was measured. Data are shown as mean ± standard error of the mean of at least three independent experiments. *P < 0.05, **P < 0.01, ***P < 0.001.

**Fig 3:**
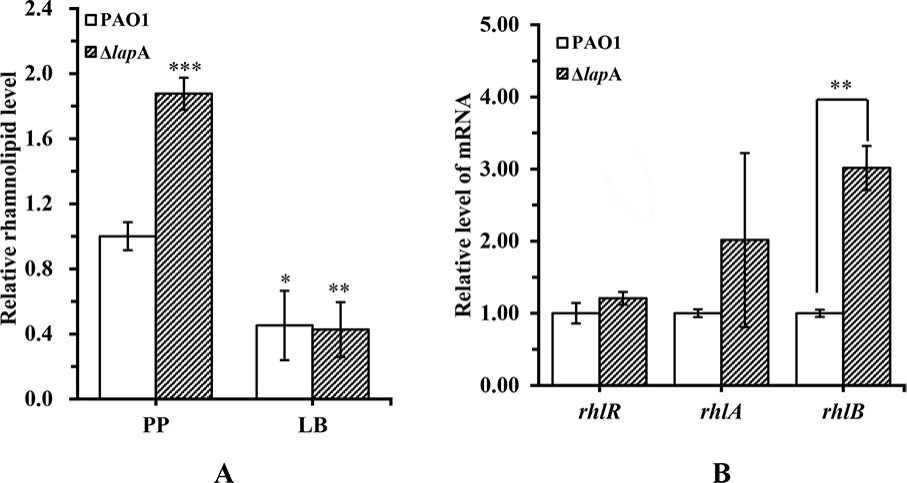
Deletion of the *lap*A gene significantly enhanced rhamnolipid production of *P. aeruginosa* PAO1 under phosphate-depleted stress. (**A**) WT and Δ*lap*A strains were cultured in phosphate-depleted and phosphate-rich media for 12 h, and rhamnolipid in the supernatants was determined. (**B**) WT and Δ*lap*A strains were incubated in the phosphate-depleted medium for 12 h, and the expression levels of *rhl*R and *rhl*A/B were measured using a quantitative reverse transcription–polymerase chain reaction. Data are shown as mean ± standard error of the mean of at least three independent experiments. *P < 0.05, **P < 0.01, ***P < 0.001.

As shown in Fig. 2A, compared with the WT strain, the amount of elastase produced by the Δ*lap*A strain was significantly reduced under phosphate depletion conditions, which was consistent with the results of the quantitative reverse transcription–polymerase chain reaction (qRT-PCR) assay. In addition, the elastase produced by the complementation strain Δ*lap*A/pLapA had a bit increase compared with the empty vector strain Δ*lap*A/pEV under phosphate-depleted conditions (Fig. S9A). On the contrary, chitinase production by the Δ*lap*A strain was significantly upregulated under phosphate-depleted conditions (Fig. 2B). However, *lap*A did not affect elastase and chitinase activities under phosphate-rich conditions. As shown in Fig. 3A, deletion of the *lap*A gene increased by 1.9 times of rhamnolipid production compared with that of the WT strain under phosphate depletion conditions, whereas it produced very less rhamnolipid under phosphate-rich conditions, which was similar to that of the WT strain. Meanwhile, the expression of genes (*rhl*R and *rhl*AB) associated with rhamnolipid synthesis was measured by qRT-PCR assay. The results showed that the expression level of *rhl*B increased by three times in the Δ*lap*A strain compared with that of the WT strain under phosphate depletion conditions (Fig. 3B). The Δ*lap*A complementation strain partially reduced rhamnolipid production compared with the control strain under phosphate depletion conditions (Fig. S9B). In addition, pyocyanin was induced in the Δ*lap*A strain in the PDB medium (specifically the pyocyanin production medium), while very less pyocyanin was produced in the PP medium, which was similar to that of the WT strain (Fig. S10). We finally investigated the effect of *lap*A on the hemolytic activity of *P. aeruginosa* but found no significant difference between both strains under the same conditions (Fig. S11).

**Fig 4:**
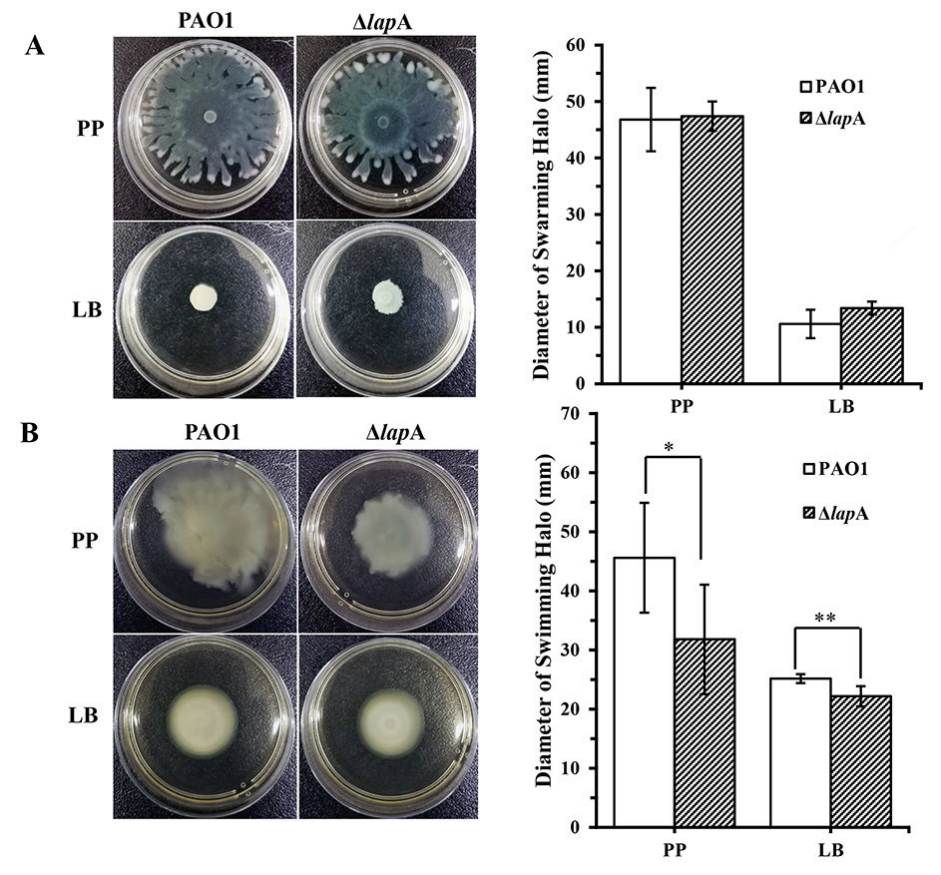
Deletion of the *lap*A gene reduced swimming motility but did not affect the swarming motility of *P. aeruginosa* PAO1 under phosphate-depleted stress. (**A**) WT and Δ*lap*A culture (1 µL) was spotted onto the swarming medium with or without phosphate and incubated for 15 h; swarming motility was evaluated, and the diameter of the halo was measured. (**B**) WT and Δ*lap*A culture (1 µL) was spotted onto the swimming medium with or without phosphate and incubated for 24 h; swimming motility was evaluated, and the diameter of the halo was measured. Data are shown as mean ± standard error of the mean of at least five independent experiments. *P < 0.05, **P < 0.01, *** *P* < 0.001.

Bacterial motility is another virulence phenotype that enhances *P. aeruginosa* infection (30, 31). We next analyzed the roles of *lap*A on swarming and swimming motilities of PAO1 under phosphate-depleted and phosphate-rich conditions. The motility assays showed that deletion of the *lap*A gene resulted in reduced swimming motility (average reduction: 30% of WT) under phosphate depletion conditions, but no significant difference was noted in swarming motility (Fig. 4; Fig. S12).

### Involvement of *lap*A in regulating the *las*/*rhl* systems

Elastase activity and rhamnolipid production are controlled by the *las* and *rhl* systems (27). Therefore, C4-HSL and 3-oxo-C12-HSL were measured using the AHL reporter plate bioassay (*Chromobacterium violaceum* CV026 as the reporter strain) and HPLC assays. Subsequently, the expression levels of *las*I, *las*R, *rhl*I, and *rhl*R were investigated using qRT-PCR assay.

**Fig 5:**
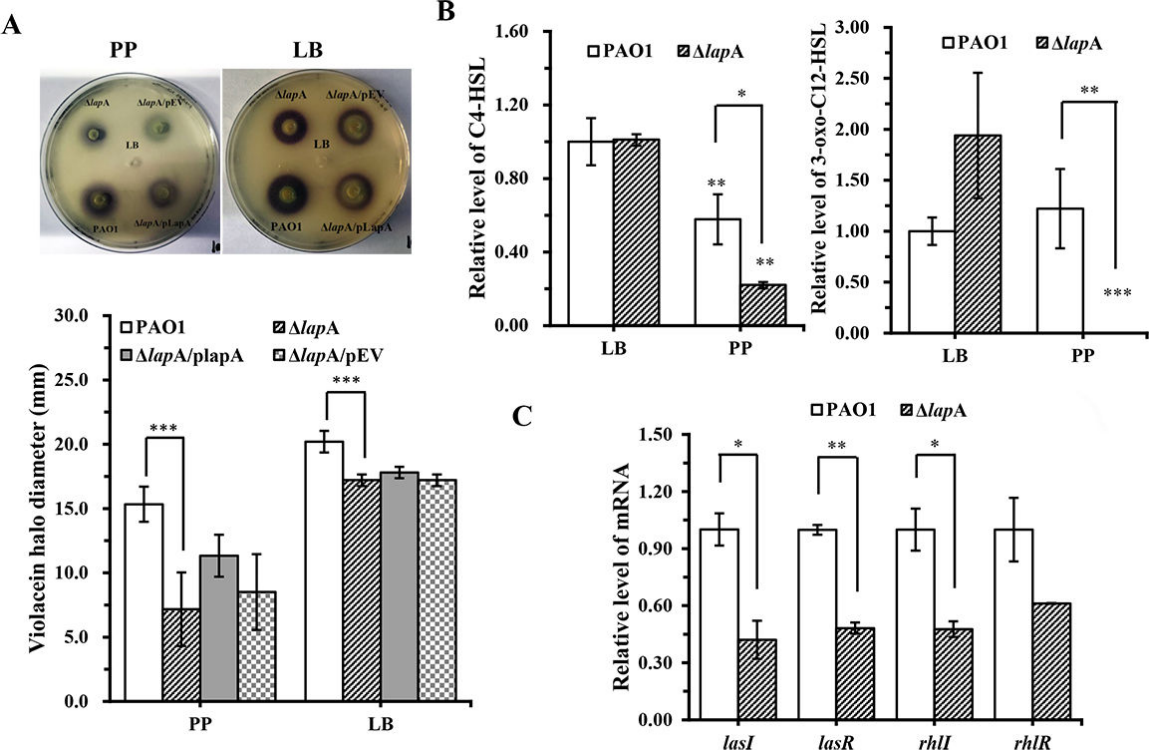
Significant regulations of the *las* and *rhl* systems of the *lap*A gene of *P. aeruginosa* PAO1 under phosphate-depleted stress. (**A**) WT, Δ*lap*A, Δ*lap*A/pLapA, and Δ*lap*A/pEV strains were added to the wells of phosphate-depleted or phosphate-rich agar plates containing *C. violaceum* CV026 and incubated at 28°C for 48 h; the violacein halo production was evaluated (top), and the diameter of the halo was measured (bottom). (**B**) WT and Δ*lap*A strains were incubated in phosphate-depleted and phosphate-rich media for 18 h. C4-HSL and 3-oxo-C12-HSL in the supernatants were extracted and measured using HPLC. The left panel presents the relative quantification of C4-HSL levels, and the right panel presents the relative quantification of 3-oxo-C12-HSL levels. All HPLC graphs are shown in Fig. S14. (**C**) WT and Δ*lap*A strains were incubated in the phosphate-depleted medium for 18 h, and the expression levels of *las*I/R and *rhl*I/R were measured using quantitative reverse transcription-polymerase chain reaction. Data are shown as mean ± standard error of the mean of at least three independent experiments. *P < 0.05, **P < 0.01, ***P < 0.001.

The AHL reporter plate bioassay showed that the production of violacein halo by the Δ*lap*A strain was significantly decreased and that by the complementary strain was increased when incubated under phosphate depletion conditions (Fig. 5A). When the strains were incubated in a phosphate-rich medium, the diameter of the violacein halo produced by the Δ*lap*A strain was significantly smaller than that of the WT strain (Fig. 5A). These results indicated that deletion of the *lap*A gene reduces C4-HSL production. The results from HPLC detection assays showed that *lap*A deletion reduced C4-HSL and 3-oxo-C12-HSL production under phosphate-depleted conditions, whereas more AHL signals were produced by both strains in the phosphate-rich medium, with an insignificant difference (Fig. 5B). Next, the effects of *lap*A on the expression of QS genes (*las*I/R and *rhl*I/R) were measured. The Δ*lap*A and WT strains were cultured in fresh PP medium for 18 h; then, the expression levels of these genes were detected by qRT-PCR assay. For *las*I, *las*R, and *rhl*I, the expression was downregulated in the Δ*lap*A strain. For *rhl*R, although deletion of the *lap*A gene downregulated its expression, no significant difference was found (Fig. 5C). Taken together, these results indicated that deletion of the *lap*A gene downregulated *las* and *rhl* expression and, consequently, decreased 3-oxo-C12-HSL and C4-HSL production, finally reducing the virulence of *P. aeruginosa* under phosphate depletion conditions. Importantly, the deletion of the *lap*A gene increased rhamnolipid production but reduced the level of C4-HSL under phosphate-depleted stress. These data suggest that rhamnolipid production would be unrelated to the C4-HSL signal under phosphate-depleted stress.

### Deletion of the *lap*A gene inhibited the biofilm formation of *P. aeruginosa* in porcine skin explants

**Fig 6:**
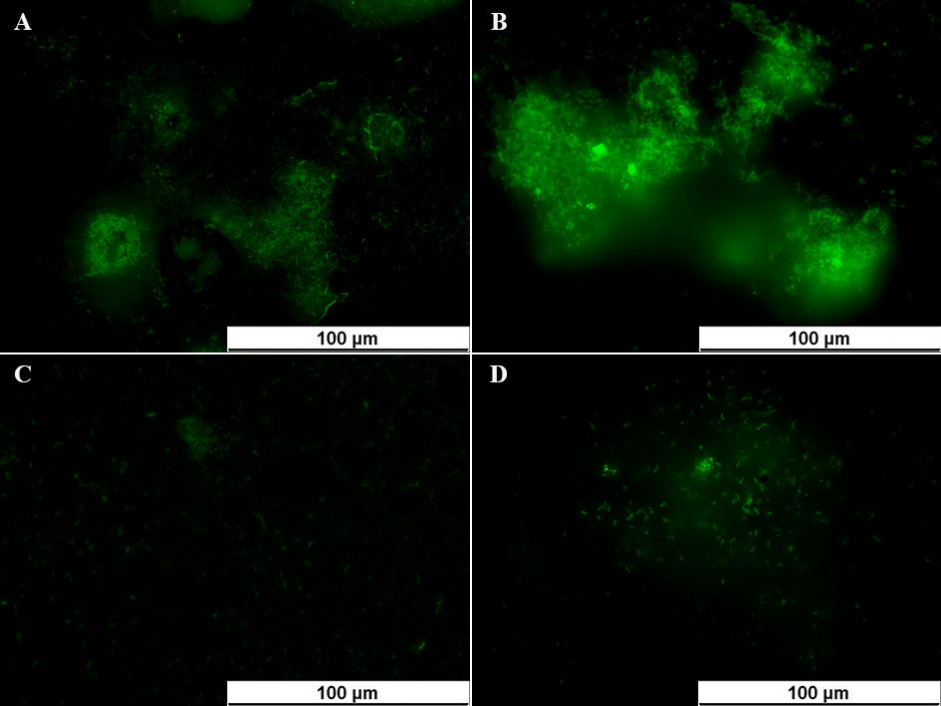
Deletion of the *lap*A gene inhibited *P. aeruginosa* PAO1 biofilm formation in porcine skin explants. (**A**) WT strain was incubated for 24 h. (**B**) The WT strain was incubated for 48 h. (**C**) The Δ*lap*A strain was incubated for 24 h. (**D**) The Δ*lap*A strain was incubated for 48 h.

As shown in our previous study, both the *lap*A gene expression level and alkaline phosphatase activity were very high in the mature biofilms formed in the *ex vivo* chronic skin wound model, a finding that is contrary to that observed in the planktonic state, early biofilm, and dispersal biofilm (22). Therefore, deletion of the *lap*A gene in the PAO1 strain was established, and its ability to form biofilms in the *ex vivo* chronic wound model was assessed. Indeed, the WT strain formed micro-colonies at 24 h and developed a biofilm that had a mushroom-like structure at 48 h. However, a few Δ*lap*A cells were attached to the porcine skin explants until 48 h (Fig. 6). Thus, the deletion of the *lap*A gene inhibited *P. aeruginosa* biofilm formation in chronic skin wounds. However, the deletion of *lap*A gene did not affect on biofilm formation of *P. aeruginosa* PAO1 *in vitro* under phosphate depletion condition and phosphate-rich conditions (Fig. S13).

**Fig 7:**
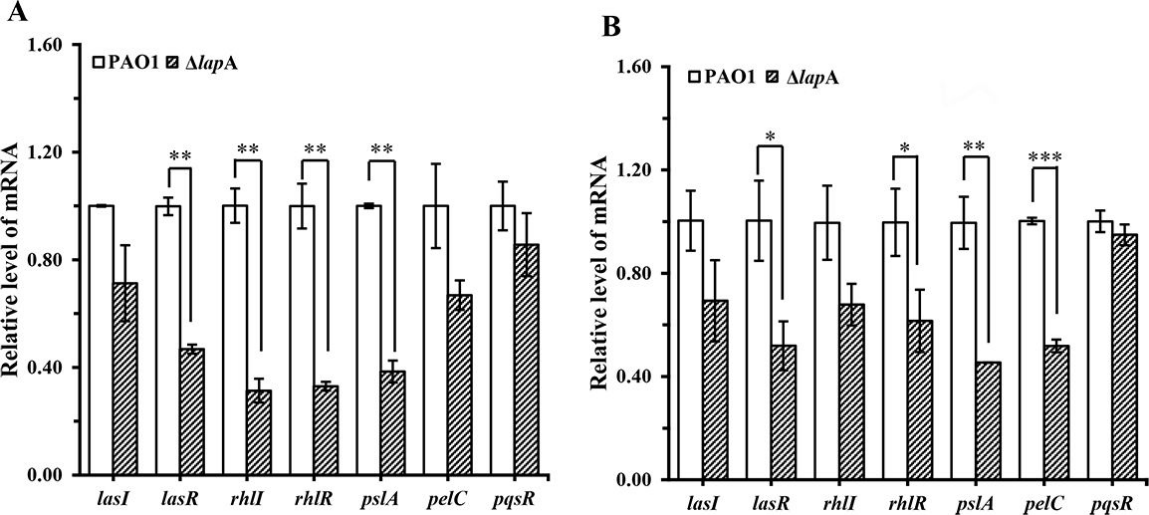
The *lap*A gene showed significant regulations in the *las* and *rhl* systems and EPS production of *P. aeruginosa* PAO1 in porcine skin explants. WT and Δ*lap*A strains were cultured in wells containing porcine skin explants and incubated for 24 and 48 h, and the expression levels of genes related to quorum sensing systems and EPS production in *P. aeruginosa* were measured using quantitative reverse transcription-polymerase chain reaction. (**A**) Relative expression levels of these genes when the WT and Δ*lap*A strains were incubated for 24 h. (**B**) Relative expression levels of these genes when the WT and Δ*lap*A strains were incubated for 48 h. Data are shown as mean ± standard error of the mean of at least three independent experiments. *P < 0.05, **P < 0.01, ***P < 0.001.

Biofilm formation by *P. aeruginosa* is positively regulated by QS systems and c-di-GMP levels. Moreover, extracellular polymeric substance (EPS), which is an indirect measurement of c-di-GMP levels, plays an important role in biofilm formation and development (32). Thus, the transcription of genes associated with QS systems and EPS was evaluated by the qRT-PCR assay to investigate the mechanism underlying the inhibition of *P. aeruginosa* biofilm formation owing to the deletion of the *lap*A gene in chronic skin wounds. The expression of the genes *las*I, *las*R, *rhl*I, and *rhl*R was downregulated in biofilms formed by the Δ*lap*A strain compared with that of the WT strain under the same conditions (Fig. 7). However, for *pqs*R, deletion of the *lap*A gene did not change its expression. Moreover, the expression levels of two important genes, namely, *psl*A and *pel*C, related to the *P. aeruginosa* biofilm Psl and Pel matrices, were investigated using the qRT-PCR assay. The results showed that the transcription levels of *pls*A and *pel*C were significantly reduced in biofilms formed by the Δ*lap*A strain compared with those of the WT strain (Fig. 7). This suggested that deletion of the *lap*A gene inhibited *P. aeruginosa* attachment to the wound bed. In summary, these results indicate that deletion of the *lap*A gene reduces biofilm formation of *P. aeruginosa* in porcine skin explants through decreases in the *las* and *rhl* QS systems and EPS synthesis.

### Deletion of the *lap*A gene reduced the virulence of *P. aeruginosa* to *C. elegans*

Our data indicated that deletion of the *lap*A gene reduced the virulence of *P. aeruginosa* under phosphate depletion conditions and inhibited biofilm formation in a chronic wound model. Therefore, we further investigated the effects of *lap*A on the pathogenicity of *P. aeruginosa* to *C. elegans*.

**Fig 8:**
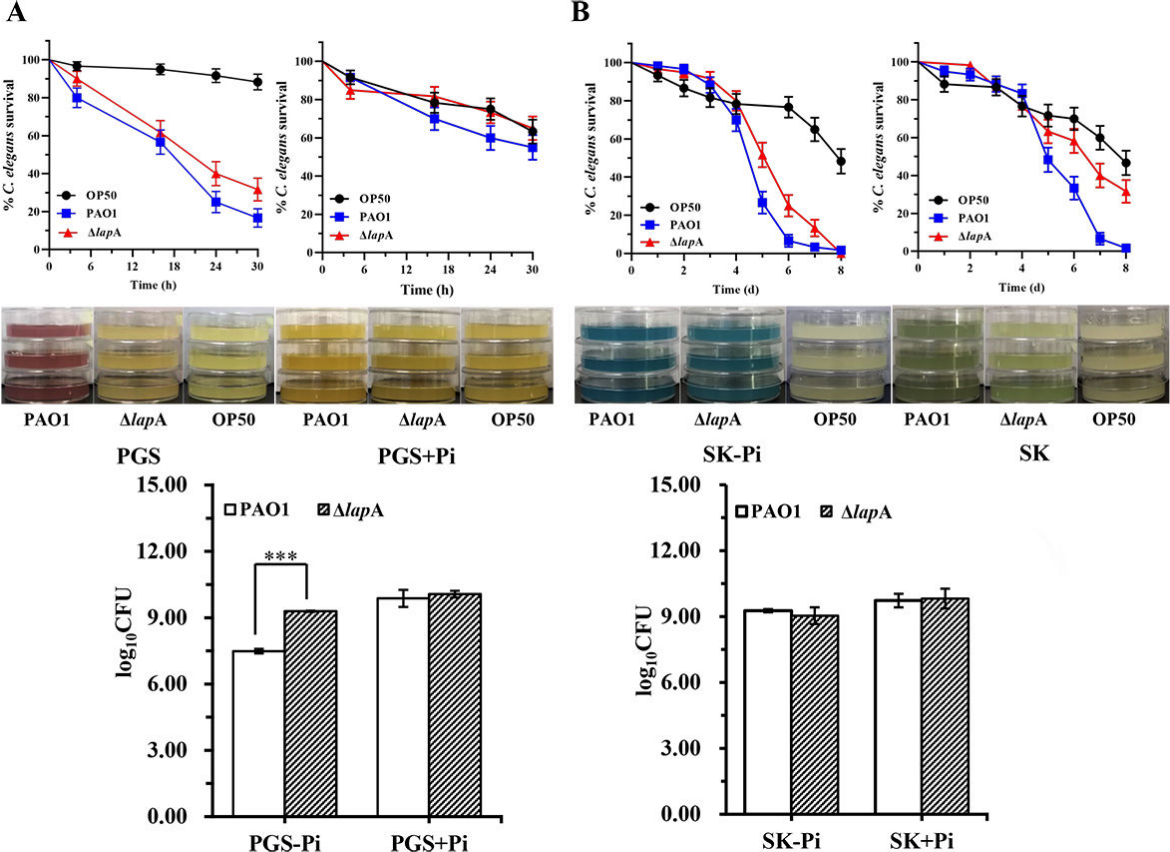
Deletion of the *lap*A gene reduced the virulence of *P. aeruginosa* PAO1 in *C. elegans* fast-kill and slow-kill infection models. (**A**) *C. elegans* was applied to lawns of PAO1 and Δ*lap*A incubated in the PGS or PGS +Pi medium for 30 h (fast-kill infection). Kaplan-Meier curves of the results are represented and compiled from three independent experiments (top). Colors of the plates containing PAO1 and Δ*lap*A strains in the PGS or PGS +Pi medium incubated at 37°C for 24 h and then at 25°C for 24 h (middle). Bacterial burden recovered from the PGS and PGS +Pi medium at 37°C for 24 h and then at 25°C for 24 h (bottom). (**B**) *C. elegans* was applied to lawns of PAO1 and Δ*lap*A incubated in the slow-killing medium without phosphate (SK-Pi medium) or SK medium rich in phosphate (SK medium) for 8 days (slow-kill infection). Kaplan-Meier curves of the results are represented and compiled from three independent experiments (top). Colors of plates containing PAO1 and Δ*lap*A in the SK-Pi or SK medium incubated at 37°C for 24 h and then at 25°C for 24 h (middle). Bacterial burden was observed in the SK-Pi or SK medium at 37°C for 24 h and then at 25°C for 24 h (bottom). Data are shown as mean ± standard error of the mean of at least three independent experiments. *P < 0.05, **P < 0.01, ***P < 0.001.

For fast-kill infection, the Δ*lap*A strain was completely avirulent under phosphate-rich conditions. Under phosphate-depleted stress, although Δ*lap*A remained virulent, it showed a slight reduction in virulence compared with the WT strain (Fig. 8A). In addition, the color of the plates containing the PAO1 strain was deeper than that of plates containing the Δ*lap*A strain in the PGS medium (Fig. 8A). The bacterial counting assay results showed that the number of visible Δ*lap*A strains was 10^9^ colony-forming units (CFUs) but 10^7^ CFU in PAO1 plates containing the PGS medium, but no difference was found in the PGS + Pi medium (Fig. 8A). For SK infection, deletion of the *lap*A gene led to a slight increase in worms’ survival rate in the SK-Pi medium. Surprisingly, the survival rate of worms was increased by 50% when they were fed on the Δ*lap*A strain for 7 days in the SK medium, whereas only 5% of the survival rate was obtained when they were fed on the WT strain under the same conditions (Fig. 8B). Therefore, the results demonstrate that the deletion of *lap*A reduces the virulence of *P. aeruginosa* to *C. elegans*.

## DISCUSSION

*P. aeruginosa* is one of the most common nosocomial pathogens that cause lethal sepsis in burn injury, major surgery, bone marrow transplantation, traumatic burns, and cystic fibrosis. Different sets of virulence factors are produced by *P. aeruginosa* during infection, such as exotoxin A, elastase, rhamnolipid, phenazines, hemolysis, swarming and swimming motilities, and biofilms (27). Of these, biofilms have been considered the main reason for chronic infection, such as cystic fibrosis and chronic wound infection (33, 34). Moreover, most of these virulence factors are controlled by a sophisticated hierarchical QS system composed of regulators with respond to cognate signal molecules. In *P. aeruginosa*, the *las* and *rhl* are the two most important QS systems which was identified two decades ago (35, 36). Besides, the production of these virulence factors is also regulated by environmental cues, such as iron and phosphate starvation (37 – 39). Generally, phosphate limitation is a common stress that *P. aeruginosa* faces when establishing an infection, as the availability of phosphate in a healthy person is low (1.25 mM) and is even lower (<0.03 mM) in patients on chemotherapy or who have undergone surgery (18). Therefore, phosphate, an essential nutrient, has been recognized as an important signal that affects virulence in *P. aeruginosa*. Previous studies demonstrated that phosphate depletion induced elastase activity, pyocyanin, and rhamnolipid production by *P. aeruginosa* by inducing the production of cell-cell signal molecules (26, 40). However, the effects of phosphate depletion on *P. aeruginosa* virulence and biofilm formation were not systematically investigated.

In this study, we demonstrate that phosphate-depleted stress regulates a wide range of virulence phenotypes of *P. aeruginosa*, including elastase and chitinase activities, rhamnolipid and pyocyanin production, hemolysis, and swarming and swimming motilities. Importantly, we have evidenced that C4-HSL does not play a key role in virulence production under phosphate-depleted stress, especially for rhamnolipid hyper-production. Previous studies indicated that phosphate-depleted stress activates *pho*BR and then induces *rhl*R expression, resulting in an increase in rhamnolipid production (26). Therefore, it is not surprising that, although the level of C4-HSL was reduced, rhamnolipid production was increased under phosphate limitation conditions.

Accumulating evidence indicates that phosphate depletion that occurs after acute surgical injuries could exacerbate the virulence of *P. aeruginosa* (21, 40). Therefore, in this study, the effects of phosphate-depleted stress on the virulence of *P. aeruginosa* in *C. elegans* were assessed in fast-kill and SK infection assays. The results of infection assays indicated that virulence was induced under phosphate-depleted stress and the pH of the culture plates dropped from approximately pH 6 before bacterial growth to pH 5 after the growth of PAO1, with the number of visible strains being reduced by 2 log. Cezairliyan et al. demonstrated that phenazine-1-carboxylic acid was the primary toxin responsible for *P. aeruginosa*-mediated killing of *C. elegans* for fast-kill infection assay (29). Therefore, in combination with that study, during inflammation, the pH of wounds with acute infection drops below pH 6, and phenazine-1-carboxylic acid exerts toxic effects in acute wound infection, in addition to other virulence components produced by *P. aeruginosa*. The results from SK infection assays indicated that pyocyanin was the key virulence component that killed worms, and the pH increased to a range of 7–8. Notably, during the early stage of wound healing, the pH of wounds increases to a range of 7–8, and pyocyanin has been shown to inhibit wound repair, which results in chronic wound infection (29). Thus, biofilm and pyocyanin may play main roles during chronic wound infection, which is difficult to heal.

Bacterial biofilm formed in the wound bed, which led to the transition of the wounds from acute to chronic infection. Once the biofilm was formed, the metabolism of bacteria within the biofilm was reduced, resulting in an increase in resistant antibiotics and immunity. Therefore, how to inhibit biofilm formation is a big clinical challenge. Our previous study indicated that the expression of *lap*A, encoding alkaline phosphate, was induced in the PAO1 biofilm formed in porcine skin explants, which is a chronic skin wound model (22). Meanwhile, our data suggested that alkaline phosphatase activity was enhanced when PAO1 formed biofilm in the *ex vivo* model (22). These results indicate that LapA would play an important role in biofilm formation in chronic wounds. Ball et al. demonstrated that alkaline phosphatase LapA was induced at phosphate-depleted stress and secreted by Hxc-T2SS (23, 24). However, the effects of the *lap*A gene on the pathogenicity of *P. aeruginosa* have not been investigated. Therefore, in this study, deletion of the *lap*A gene in PAO1 was established to investigate the effects of *lap*A on the virulence and biofilm formation of *P. aeruginosa*. Significantly, deletion of the *lap*A gene reduced the virulence of *P. aeruginosa* under phosphate-depleted conditions, including elastase activity and swimming motility. On the contrary, *lap*A mutation enhanced rhamnolipid production under phosphate-depleted stress. However, no difference was found when both strains were incubated in a phosphate-rich medium. As known, elastase activity and rhamnolipid production are controlled by the *las* and *rhl* systems in *P. aeruginosa* (14, 27, 40). Therefore, the expression levels of *las*I/R and *rhl*I/R were measured using qRT-PCR assay. The results indicated that deletion of the *lap*A gene reduced the transcription of the genes *rhl*I, *las*I, and *las*R, with no difference in the expression of *rhl*R under phosphate-depleted stress. Subsequently, 3-oxo-C12-HSL and C4-HSL were tested using an HPLC system. The results indicated that the levels of both components were reduced in the Δ*lap*A strain compared with those of the WT strain under phosphate-depleted stress, consistent with the results of the qRT-PCR assay. Therefore, deletion of the *lap*A genes reduced the elastase activity owing to the inhibition of the *las* system. Although C4-HSL was reduced when *lap*A was deficient, rhamnolipid production was induced under phosphate-depleted conditions, which further suggests that C4-HSL was unrelated to rhamnolipid production under phosphate-depleted stress. Indeed, previous studies indicated that the two-component regulatory system PhoRB upregulated RhlR expression, resulting in the hyper-production of rhamnolipid, which had no effect on C4-HSL (26, 40). Therefore, it is not surprising that the C4-HSL level was reduced, but rhamnolipid production was increased.

Phosphate depletion occurs after a surgical injury, which significantly increases the virulence of *P. aeruginosa* (5, 21). Biofilm formation is a key example of *P. aeruginosa* community behavior that is regulated by QS (35, 36, 41). Our previous data provided evidence that porcine skin explants can provide a phosphate-depleted environment and mimic the actual skin tissue. Therefore, although the model used in this study did not show any immune response to chronic wounds, the model could mimic the tissue in a better way than that of other *in vitro* models. In this study, our data indicated that deletion of the *lap*A gene inhibited *P. aeruginosa* biofilm formation in the *ex vivo* model. To better understand the involvement of *lap*A in biofilm formation and QS systems, we investigated the expression levels of *las*I/R, *rhl*I/R, and *pqs*R in both Δ*lap*A and WT strains when they formed biofilms in porcine skin explants. Compared with the WT strain, our data (Fig. 7) suggest that *lap*A may have a stronger influence on the *las* and *rhl* systems but had no significant influence on the *pqs* system, which was consistent with our results for the PP medium, a phosphate-depleted medium. EPS is a major component of the biofilm matrix. *P. aeruginosa* PAO1 primarily relies on Psl and Pel for biofilm formation (32, 42). Both Psl and Pel play an important role in the surface adherence of the biofilm, including adherence on the surfaces of glass, polyvinyl chloride, mucin, and epithelial cells (43 – 45). Ghafoor et al. demonstrated that mutants deficient in both Psl and Pel production lost their ability to form biofilms (46). Therefore, Psl and Pel would be potential targets for inhibiting *P. aeruginosa* biofilm. In our work, the results of qRT-PCR assays demonstrated that the expression levels of *psl*A and *pel*C were reduced in the Δ*lap*A strain compared with those in the WT strain. These results suggest that deletion of the *lap*A gene would inhibit *P. aeruginosa* biofilm formation in porcine skin explants through inactivation of the *las* and *rhl* QS systems and a decrease in EPS production.

In summary, this study conducted a systematic investigation of the effect of phosphate-depleted stress on the virulence of *P. aeruginosa* through *in vitro*, *ex vivo*, and *in vivo* experiments. These results showed that phosphate-depleted stress could enhance elastase activity, hemolysis, rhamnolipid production, swarming and swimming motilities, and 3-oxo-C12-HSL production, while the production of these virulence factors was unrelated to C4-HSL signals under phosphate-depleted stress. Meanwhile, phosphate-depleted stress can increase the lethal ratio of worms by inducing phenazine-1-carboxylic acid for fast-kill infection and pyocyanin for SK infection. Importantly, we observed that deletion of the *lap*A gene, encoding alkaline phosphatase, reduced elastase activity, swimming, C4-HSL, and 3-oxo-C12-HSL production of PAO1, while it increased chitinase activity and rhamnolipid production under phosphate-depleted stress. In addition, deletion of the *lap*A gene significantly inhibits PAO1 biofilm formation in porcine skin explants, a chronic wound model, by reducing the expression levels of the *las* and *rhl* QS systems and EPS synthesis. Therefore, these findings emphasize the importance of *lap*A for virulence and biofilm formation by *P. aeruginosa* under phosphate-depleted stress. Gaining an understanding of these environmental cues and the underlying regulatory mechanisms may aid in developing effective therapies against bacterial infection. Phosphate supplementation has been proven effective in the reduction of virulence caused by *P. aeruginosa* in intestinal and burn wound infections. Meanwhile, LapA has been proven as a target to reduce virulence production and inhibit biofilm formation by *P. aeruginosa* in chronic wounds. Thus, this study reports targeting LapA as a potential approach to control biofilm formation and reduce virulence in nonhealing-infected skin wounds, wherein *P. aeruginosa* biofilms are a persistent problem.

## MATERIALS AND METHODS

### Bacterial strains, plasmids, and growth conditions

The bacterial strains and plasmids used in the present study are listed in Table S1. Unless otherwise indicated, all strains were cultured in LB (Miller; containing 10 g of tryptone, 10 g of NaCl, and 5 g of yeast extract per liter) at 37°C under shaking at 150 rpm. PP medium containing 0.4% glucose was used as the phosphate-limiting medium, and LB broth was used as the phosphate-rich medium (23, 24). The following antibiotics were used at the indicated concentrations as needed: for *P. aeruginosa* strains, tetracycline 50 µg/mL and gentamicin 50 µg/mL; for *Escherichia coli* strains, tetracycline 15 µg/mL, gentamicin 50 µg/mL, nalidixic acid 50 µg/mL, and apramycin 50 µg/mL. All antibiotics were purchased from Adamas (Shanghai, China).

### Construction of *lap*A-deficient and complementation strains

The *lap*A gene was inactivated by insertional inactivation through double-crossover homologous recombination according to the method described by Park et al. with slight modifications (47). Briefly, the pXT01 knockout plasmid based on pKC1139 was constructed by amplifying the tetracycline resistance gene as a selection marker from the pCasPA plasmid and left-and-right-flaking regions of the *lap*A gene using the genomic DNA of PAO1 as a template. The primer pairs *lap*A-P1-P2, *lap*A-P3-P4, and *tet*R-P1-P2 (Table S2) were designed for the amplification of left-and-right-flaking fragments of the *lap*A gene and selection marker. DNA assembly was performed by digestion using restriction enzymes (Thermo Fisher Scientific, USA) and ligation with T4 DNA ligase (New England Biolabs, England) according to the manufacturer’s instructions. The pXT01 plasmid was passaged through *E. coli* S17–1λ-pir and then introduced into the PAO1 strain by conjugation (48). The target region of the *lap*A gene was then disrupted by insertional inactivation through double-crossover homologous recombination. The desired mutant Δ*lap*A was selected based on its tetracycline-resistant and apramycin-sensitive phenotype and verified by PCR using the primer pair *lap*A-P5-P6 (Table S2), after which it was identified by sequencing. The resulting *lap*A deletion mutant of PAO1 was designated as Δ*lap*A.

The empty vector pBBR1MCS-5, a gentamicin-resistant broad-host cloning vector, was used to construct the Δ*lap*A complementation strain. The whole sequence of the *lap*A gene was amplified by PCR using the primer pair *lap*A-P7-P8 (Table S2), wherein the genomic DNA of PAO1 was used as the template. The PCR product and the empty vector pBBR1MCS-5 were digested with the restriction enzymes EcoRI and BamHI (Thermo Fisher Scientific, USA), respectively, according to the manufacturer’s instructions. Next, the digested products were purified and ligated using T4 DNA ligase (New England Biolabs, England) according to the manufacturer’s protocols. The ligation product was chemically transformed into *E. coli* DH5α competent cells, and the transformed cells were plated onto LB agar plates containing gentamicin. The recombinant plasmid was identified using PCR assay, after which it was digested using EcoRI and BamHI and then sequenced. Finally, the target plasmid, named pLapA, was chemically transformed into the Δ*lap*A strain. The Δ*lap*A strain containing the plasmid pLapA was named the Δ*lap*A/pLapA strain. Meanwhile, an empty plasmid pBBR1MCS-5 was transformed into the Δ*lap*A strain as the control strain, named the Δ*lap*A/pEV strain.

### Alkaline phosphatase activity assays

A single colony from the LB plates containing the culture of PAO1 and Δ*lap*A strains was inoculated into 2 mL of LB medium and incubated at 37°C overnight. Then, 5 µL of each overnight culture was added to 5 mL of fresh PP medium or LB medium and then incubated at 37°C under shaking at 150 rpm for 4, 12, and 18 h. Then, 200 µL of each culture was taken, and the absorbance was measured at OD_600_ using a microplate reader (TECAN Spark, Switzerland). In addition, 1 mL of each culture was taken and centrifuged for 5 min at 10,000 rpm. The supernatants were collected and passed through a 0.22-µm syringe filter, and alkaline phosphatase activity was detected in the filtrate using the Alkaline Phosphatase Assay Kit (Beyotime, Beijing, China) according to the manufacturer’s instructions. The results were normalized to OD_600_.

### Virulence factor assays

#### Pyocyanin production assay

This assay was performed according to the method described by Mukherjee et al. with slight modifications (14, 49). Briefly, 5 µL of each overnight culture was added to 5 mL of fresh PP medium or PDB medium and incubated at 37°C under shaking at 150 rpm for 18 h. The supernatant was collected by centrifugation, which was then passed through a 0.22-µm filter and collected into clear glass tubes. Then, 200 µL of each filtered supernatant was transferred into a new 96-well plate (Corning 3599, USA) and the OD_695_ of each sample was measured using a microplate reader (TECAN Spark, Switzerland). In addition, the OD_600_ of each culture was measured using the same microplate reader.

#### Elastase assay

Briefly, 5 µL of each overnight culture was added to 5 mL of fresh PP medium or LB medium and incubated at 37°C under shaking at 150 rpm for 18 h. The supernatant was collected by centrifugation, and elastase activity was measured using the EnzChek Elastase Assay Kit (Invitrogen, MA, USA) according to the manufacturer’s instructions. The kit consists of the BODIPY fluorophore (FL)–labeled DQ elastin conjugate as a substrate of elastase. The BODIPY FL-labeled DQ elastin conjugate, when cleaved by the elastase enzyme, yields highly fluorescent fragments. Fluorescence was recorded every 10 min for 2 h using a microplate reader (TECAN Spark, Switzerland), with the excitation and emission wavelengths being at 490 and 520 nM, respectively.

#### Chitinase activity assay

Briefly, 5 µL of each overnight culture was added to 5 mL of fresh PP medium or LB medium and incubated at 37°C under shaking at 150 rpm for 18 h. Chitinase activity was measured using the Micro Chitinase Assay Kit (Solarbio Life Sciences, Beijing, China) according to the manufacturer’s instructions. Finally, 200 µL of the reaction solution was added to a new 96-well plate, and the OD_585_ of each sample was measured using a microplate reader (TECAN Spark, Switzerland).

#### Hemolysis assay

The assay was performed according to the method described by Pu et al. with slight modifications (27). Briefly, 5 µL of each overnight culture was added to 5 mL of fresh PP medium or LB medium and incubated at 37°C under shaking at 150 rpm for 18 h. Next, 600 µL of the indicated strain was washed once with phosphate-buffered saline (PBS) and resuspended in PBS, after which it was mixed with 600 µL of 4% sheep blood suspension in a sterile tube. The co-cultures were incubated at 37°C under shaking at 150 rpm for 4 h. Next, 200 µL of the supernatant after centrifugation was added to a new 96-well plate (Corning 3599, USA), and the OD_450_ of each sample was measured using a microplate reader (TECAN Spark, Switzerland). Meanwhile, 2% Triton X-100 was used as a positive control, and PBS was used as a negative control. The hemolysis rate (%) was calculated using the following formula: (*A*_co-culture_ − *A*_PBS_)/(*A*_Triton X-100_ − *A*_PBS_) × 100%, as described previously (27).

#### Rhamnolipid assay

Rhamnolipid production was measured by methylene blue complexation according to the methods previously described with slight modifications (50). Briefly, 5 µL of each overnight culture was added to 5 mL of fresh PP medium or LB medium and incubated at 37°C under shaking at 150 rpm for 12 h. Culture supernatant (1 mL) was acidified with 1 M HCl, and rhamnolipid was then extracted with 5 mL of chloroform. Three milliliters of the chloroform extract was taken in a new tube and then allowed to react with 100 µL of methylene blue (1 g/L) and 5 mL of distilled water. Finally, 200 µL of the chloroform layer was collected, and the OD_638_ of each sample was measured using a microplate reader (TECAN Spark, Switzerland).

### Motility assays

These assays were performed according to a previously described method with a few modifications (51). Media for swimming (PP medium containing 0.3% agar or LB medium containing 0.3% agar) and swarming (PP medium containing 0.4% agar or LB medium containing 0.4% agar) assays were prepared. Next, 1 µL of each overnight culture was spotted onto the center of a plate (60 mM diameter) containing 10 mL of each type of medium. The plates were incubated at 37°C for 24 h, after which the diameters of the motility zone developed by each strain were measured. Each experiment was repeated at least three times in triplicate.

### qRT-PCR assay

Total RNA was extracted using the Spin Column Bacterial Total RNA Purification Kit (Sango Biotech, China) according to the manufacturer’s instructions. Complementary DNA was synthesized using the MonScript RTIII Super Mix with dsDNase Kit (Monad, China) according to the manufacturer’s instructions. The primers used for this assay were designed using Primer3 software, and the sequences are listed in Table S2. qRT-PCR assay was performed in a 20 µL reaction volume using MonAmp SYBR Green qPCR Mix (Monad, China) according to the manufacturer’s instructions. These reactions were performed using the LightCycler 96 Instrument (Roche Diagnostics, USA) by applying the following cycle parameters: 95°C for 30 s, followed by 40 cycles at 95°C for 5 s, 60°C for 30 s, and 95°C for 15 s. All experiments were performed in triplicate, and measurements were recorded; the results were normalized with the housekeeping genes *rps*L and *rec*A, which were used as the internal reference genes at planktonic state and biofilms, respectively. Fold changes between the WT and mutant samples were calculated using the 2^−∆∆Ct^ method.

### AHLs detection assays

*C. violaceum* CV026 is used as a biosensor to visualize AHLs with N-acyl side chains from C4-C8 in length produced by Gram-negative bacteria (27, 52). In this study, one colony of CV026 was inoculated into 2 mL of LB medium and incubated at 28°C overnight. The CV026 culture was added to a warm PP or LB agar (1.5%) medium at a ratio of 1:100, and the mixture was then poured immediately over the surface of PP or LB agar plates prepared in Petri dishes. When the agar solidified, five wells (5 mm diameter for each well) were prepared in each plate, whose bottoms were sealed with warm agar solution. Next, 25 µL of each overnight culture was added to each well. Meanwhile, 25 µL of LB was prepared under the same conditions as those of the control. Violacein halo production was observed after incubation at 28°C for 48 h, and the diameter of each violacein halo was measured.

AHL signal molecules produced by *P. aeruginosa* were extracted and measured according to the methods described previously by our group with slight modifications (53). Briefly, AHL production was determined by inoculating 200 µL of overnight culture into 200 mL of PP or LB medium. After 18 h of cultivation at 37°C, the sterile supernatant was collected and extracted with acidified ethyl acetate according to a protocol described previously (54). AHLs produced by bacteria were analyzed using the HPLC system (Shimadzu, Japan) equipped with a C18 column by ultraviolet absorbance at 210 nm. Mobile phase A was water, and mobile phase B was methanol. The flow rate was set as 0.8 mL/min. The injection volume was 20 µL. The peaks corresponding to C4-HSL and 3-oxo-C12-HSL were identified according to the retention time of commercial C4-HSL and 3-oxo-C12-HSL standards (Aladdin, Shanghai, China) according to the same HPLC protocol.

### Biofilm formation assays

*Ex vivo* biofilm formation assay was performed according to the method previously described by our group (22). Briefly, 10 µL (10^6^ CFUs) of overnight culture was added into each porcine skin explant well. Soft agar plates were statically incubated at 37°C. All explants were transferred into fresh soft agar plates (containing only 0.5% agar) containing irgasan (25 µg/mL) each day. Then, 10 µL of LB medium was added to each explant well, and the plate was incubated under the same conditions as those of the negative control. To quantify the biofilms developed in the porcine skin explant wells, the explants were gently washed with 10 mL of sterile PBS three times to remove loosely bound cells. The explants were then sonicated in 2 mL tubes containing 1 mL of sterile PBS for 30 s, followed by vigorous mixing. Proper dilutions were made with sterile PBS and plated on *Pseudomonas* isolation agar plates. The plates were incubated at 37°C overnight, after which the bacterial colonies were counted. One set of washed explants was stained using the acridine orange/ethidium bromide staining kit (Sangon Biotech, China) according to the manufacturer’s protocol. Imaging of biofilms in explant wells was performed under a fluorescence microscope (Leica Microsystems, Germany). The imaging areas were chosen at the center of the reservoir to avoid edge effects.

*In vitro,* biofilm was measured according to the methods previously described with slight modifications (55, 56). Observation of biofilm formation was carried out in 5 mL borosilicate tubes. Briefly, overnight cultures were inoculated at 1:1,000 dilutions into 1 mL of PP or LB medium and statically grown at 37°C for 18 h. Biofilms were stained with 0.1% crystal violet and tubes were washed with water to remove unbound dye. The remaining crystal violet was dissolved in 1 mL of 95% ethanol. A 200 µL of this solution was transferred to a new 96-well plate, and the absorbance was measured at 570 nm.

### *C. elegans* slow- and fast-killing assays

*C. elegans* killing assays were performed with WT N2 worms for each condition according to previously described methods (57). *C. elegans* N2 strain was purchased from the Caenorhabditis Genetics Center (MN, USA). The worms were propagated on plates containing nematode growth medium (NGM), after which the eggs were harvested from gravid adults according to a standard bleaching protocol. The harvested eggs were plated on lawns of fresh *E. coli* OP50 and allowed to grow until they reached the L4 stage at 25°C. The worms at the L4 stage were then transferred to NGM plates without any strain.

For SK assays, 20 worms at the L4 stage were moved to lawns of WT and Δ*lap*A strains in SK assay plates with phosphate (containing peptone, 0.35%; NaCl, 0.3%; cholesterol, 5 µg/mL; agar, 2%; CaCl_2_, 1 mM; MgSO_4_, 1 mmol/L; KH_2_PO_4_, 25 mmol/L) or without phosphate. All experimental plates were supplemented with nalidixic acid (5 µg/mL) to inhibit the growth of OP50 and 5-fluoro-2′-deoxyuridine (FUDR, 25 µg/mL; Adamas, China) to inhibit egg production. The plates were incubated at 25°C, and nematodes were scored for their survival every 24 h for 8 days. At the same time, 20 worms were moved to lawns of *E. coli* OP50 on the same plates except those containing nalidixic acid and incubated under the same conditions as those of the reference.

For fast-killing assays, 20 worms at the L4 stage were moved to lawns of WT and Δ*lap*A strains in PGS agar plates (containing 1% peptone, 1% NaCl, 1% glucose, 0.15 M sorbitol, and 1.7% agar) and PGS plates supplemented 1 mM KH_2_PO_4_. All experimental plates were supplemented with nalidixic acid (5 µg/mL) to inhibit the growth of OP50 and FUDR (25 µg/mL) to inhibit egg production. Nematodes were scored for survival at time points of 4, 16, 24, and 30 h. Meanwhile, 20 worms at the L4 stage were moved to lawns of *E. coli* OP50 on the same plates without nalidixic acid and incubated under the same conditions as those of the reference.

### Statistical analysis

All experiments were performed in triplicate and repeated on different days unless otherwise stated. The results are summarized as mean ± standard deviation. One-way analysis of variance was performed together with Student’s *t*-test to determine statistically significant differences. *P* < 0.05 was considered to indicate statistical significance. The log-rank (Mantel-Cox) test was performed using Prism GraphPad (version 9) software (San Diego, CA, USA) to compare the nematode killing rate between the experimental and control groups.

## SUPPLEMENTAL MATERIAL

Supplemental figures spectrum.02060-23-s0001.pdf Fig. S1 to S13.

Fig. S14 spectrum.02060-23-s0002.pdf All HPLC graphs.

Table S1 spectrum.02060-23-s0003.docx Bacterial strains and plasmids used in this study.

Table S2 spectrum.02060-23-s0004.docx Sequences of the primers used in this study.

ASM does not own the copyrights to Supplemental Material that may be linked to, or accessed through, an article. The authors have granted ASM a non-exclusive, world-wide license to publish the Supplemental Material files. Please contact the corresponding author directly for reuse.
