# Supplementary material for: Alkaline phosphatase LapA regulates quorum sensing–mediated virulence and biofilm formation in Pseudomonas aeruginosa PAO1 under phosphate depletion stress
Source: Microbiol Spectr. 2023 Oct 5;11(6):e02060-23. doi: 10.1128/spectrum.02060-23 (PMC10715133; doi:10.1128/spectrum.02060-23)
Supplement: Supplemental figures — Fig. S1 to S13. [file spectrum.02060-23-s0001.pdf]

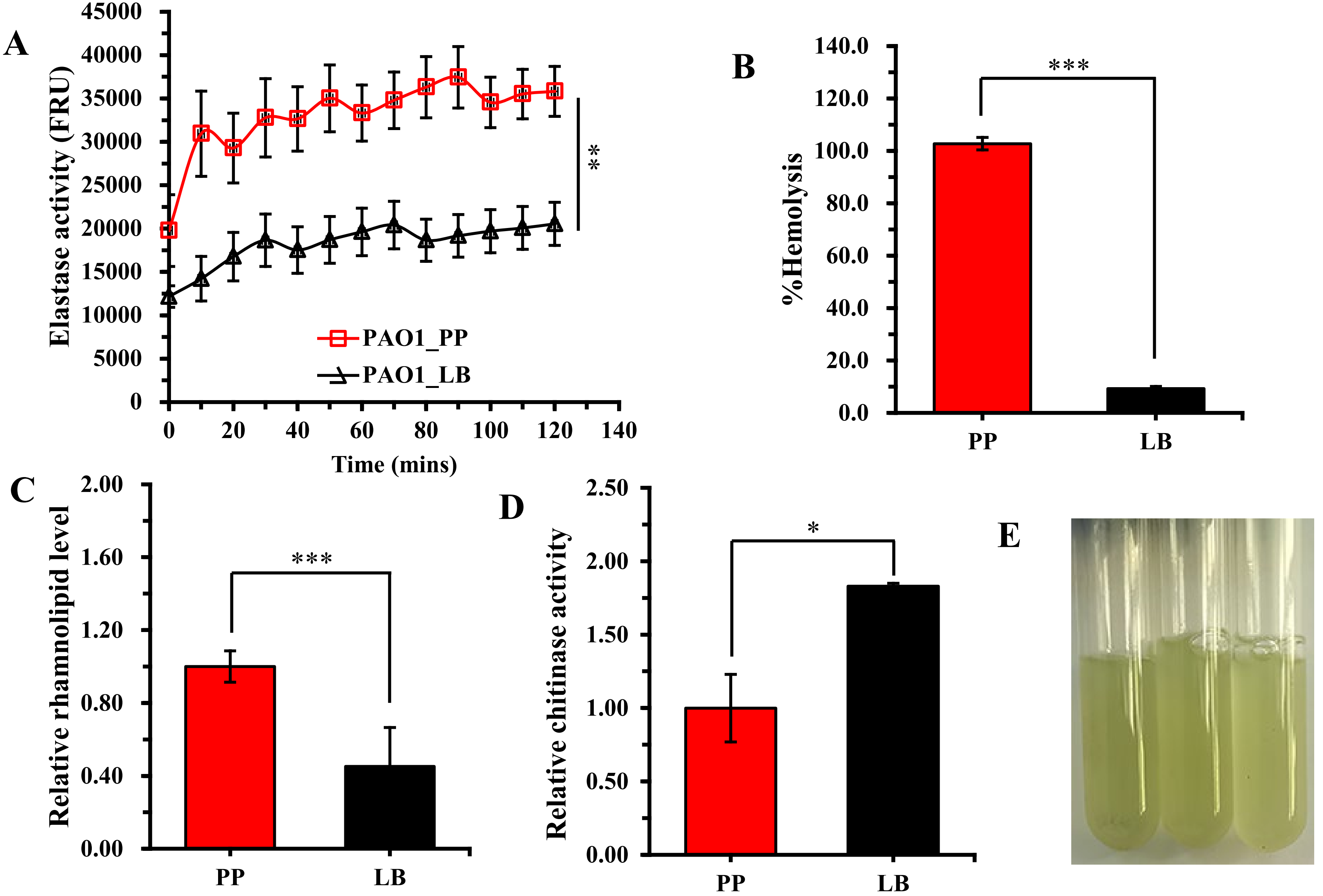

FIG S1 Phosphate-depleted stress enhanced elastase activity, hemolysis, and rhamnolipid production, but reduced chitinase activity and pyocyanin production of *Pseudomonas aeruginosa* PAO1. (A) Elastase in the supernatant of PAO1 strain that cultured in phosphate-depleted and -rich media for 18 h. (B) Hemolytic activity of PAO1 strain that cultured in phosphate-depleted and -rich media for 18 h was detected with 4% sheep blood, 2% TritonX-100 was used as a positive control, and PBS as a negative control. (C) PAO1 strain was cultured in phosphate-depleted and -rich media for 12 h, and rhamnolipid in the supernatants were determined. (D) PAO1 strain was cultured in phosphate-depleted and -rich media for 18 h, and chitinase activity in the pellets was measured. (E) Very less pyocyanin was produced in the PP medium. Data are shown as mean ±SEM of at least three independent experiments. *, $p<0.05$; **, $p<0.01$; ***, $p<0.001$.

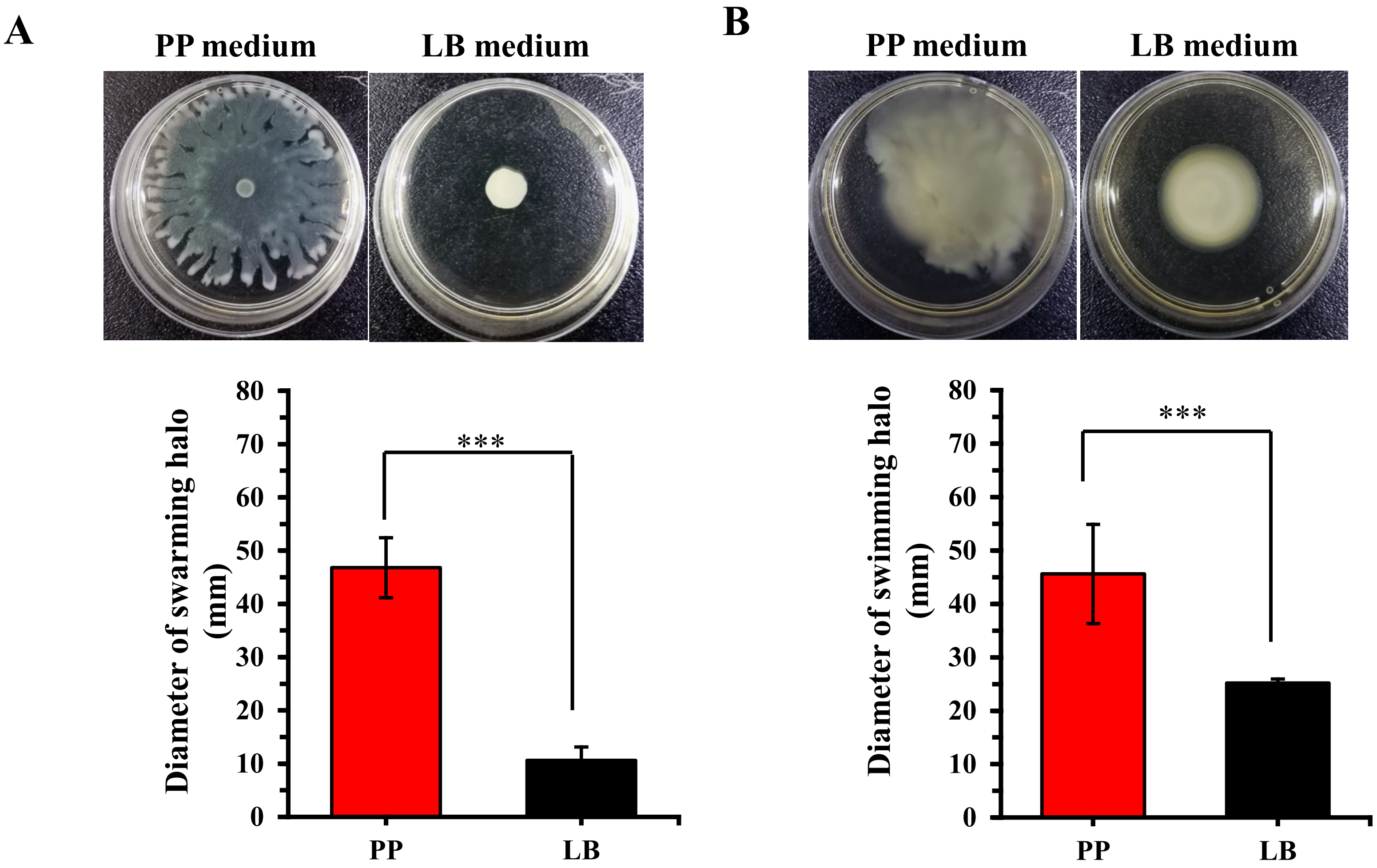

FIG S2 Phosphate-depleted stress positively regulated swarming and swimming motilities of *Pseudomonas aeruginosa* PAO1. (A) PAO1 culture (1μL) was spotted onto the swarming medium with or without phosphate, and incubated for 15 h; swarming motility was evaluated, and the diameter of the halo was measured. (B) PAO1 culture (1μL) was spotted onto the swimming medium with or without phosphate and incubated for 24 h; swimming motility was evaluated, and the diameter of the halo was measured. Data are shown as mean ±SEM of at least five independent experiments. *, $p<0.05$; **, $p<0.01$; ***, $p<0.001$.

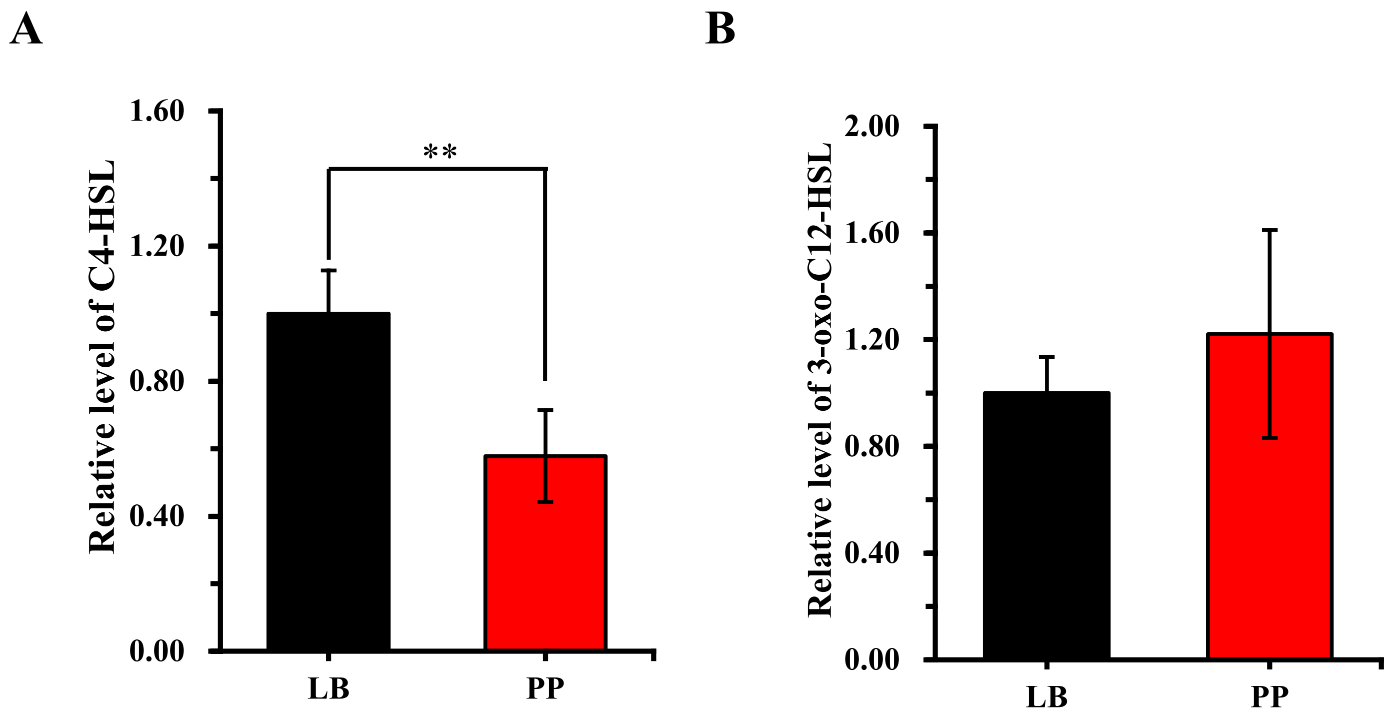

FIG S3 Phosphate-depleted stress negatively regulated C4-HSL production, but had no effect on 3-oxo-C12-HSL production of *Pseudomonas aeruginosa* PAO1. The strain was incubated in phosphate-depleted and -rich media for 18 h, respectively. C4-HSL and 3-oxo-C12-HSL in supernatants were extracted and then measured using high-performance liquid chromatography. (A) The relative quantification of C4-HSL level. (B) The relative quantification of 3-oxo-C12-HSL level. Data are shown as mean ±SEM of at least three independent experiments. *, $p<0.05$; **, $p<0.01$; ***, $p<0.001$.

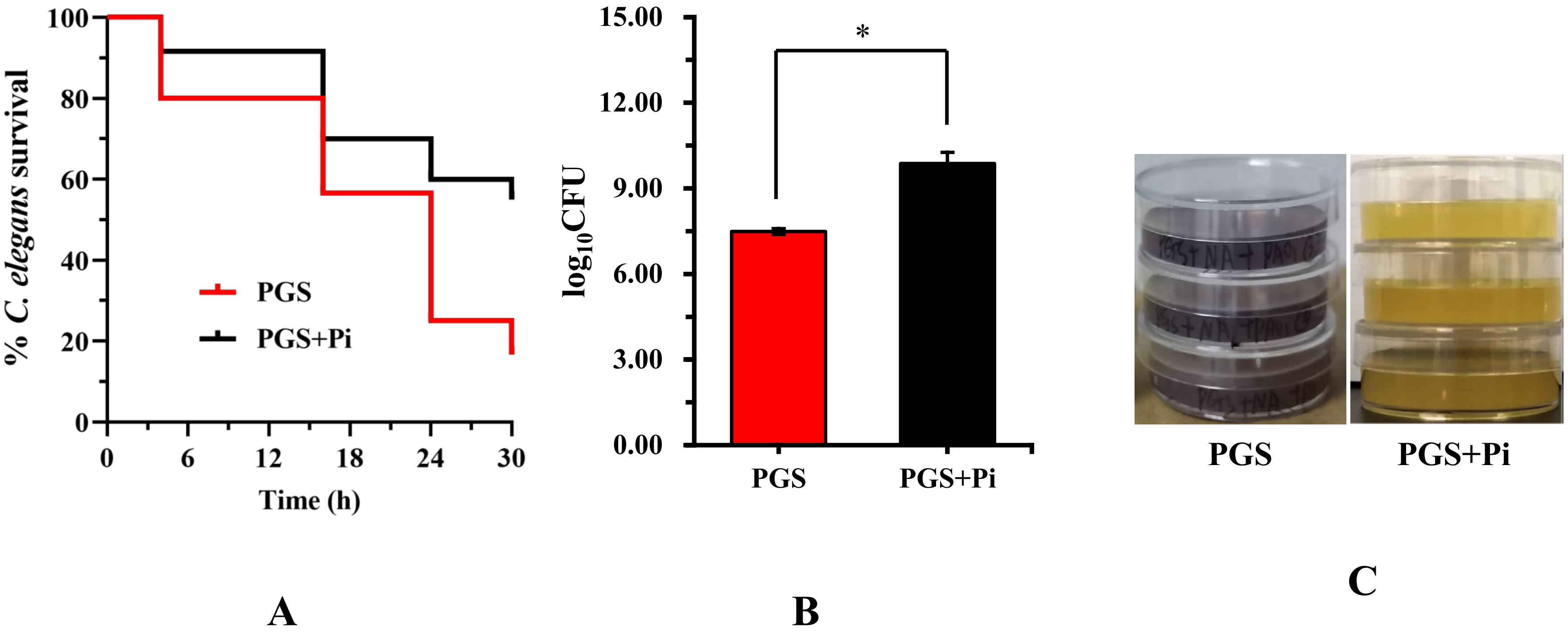

FIG S4 Phosphate depletion is required for virulence produced by *Pseudomonas aeruginosa* PAO1 in *Caenorhabditis elegans* fast-kill infection. (A) *C. elegans* were applied to lawns of PAO1 incubated in PGS or PGS+Pi medium for 30 h. Kaplan-Meier curves of the results are represented and compiled from three independent experiments. (B) Bacterial burden recovered from PGS and PGS+Pi medium at 37°C for 24 h and then at 25°C for 24 h. (C) Colors of the plates containing PAO1in PGS or PGS+Pi medium at 37°C for 24 h and then at 25°C for 24 h. Data are shown as mean ±SEM of at least three independent experiments. *, $p<0.05$; **, $p<0.01$; ***, $p<0.001$.

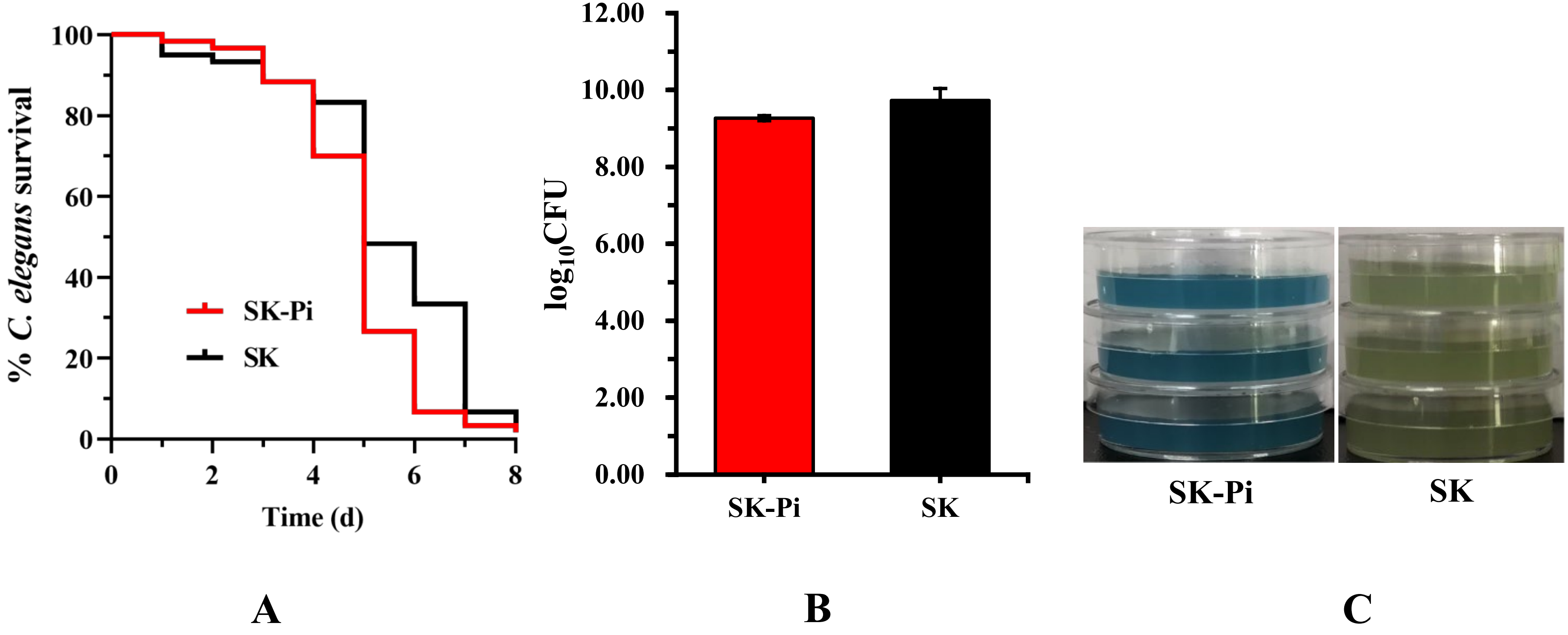

FIG S5 Phosphate depletion is required for pathogenicity of *Pseudomonas aeruginosa* PAO1 to *Caenorhabditis elegans* in slow-kill infection model. (A) *C. elegans* was applied to lawns of PAO1 incubated in the slow-killing medium without phosphate (SK-Pi medium) or SK medium rich in phosphate (SK medium) for 8 days. Kaplan-Meier curves of the results are represented and compiled from three independent experiments.(B) Bacterial burden recovered from SK-Pi or SK medium at 37°C for 24 h and then at 25°C for 24 h. (C) Colors of plates incubating PAO1 in SK-Pi or SK medium at 37°C for 24 h and then at 25°C for 24 h. Data are shown as mean ±SEM of at least three independent experiments. *, $p<0.05$; **, $p<0.01$; ***, $p<0.001$.

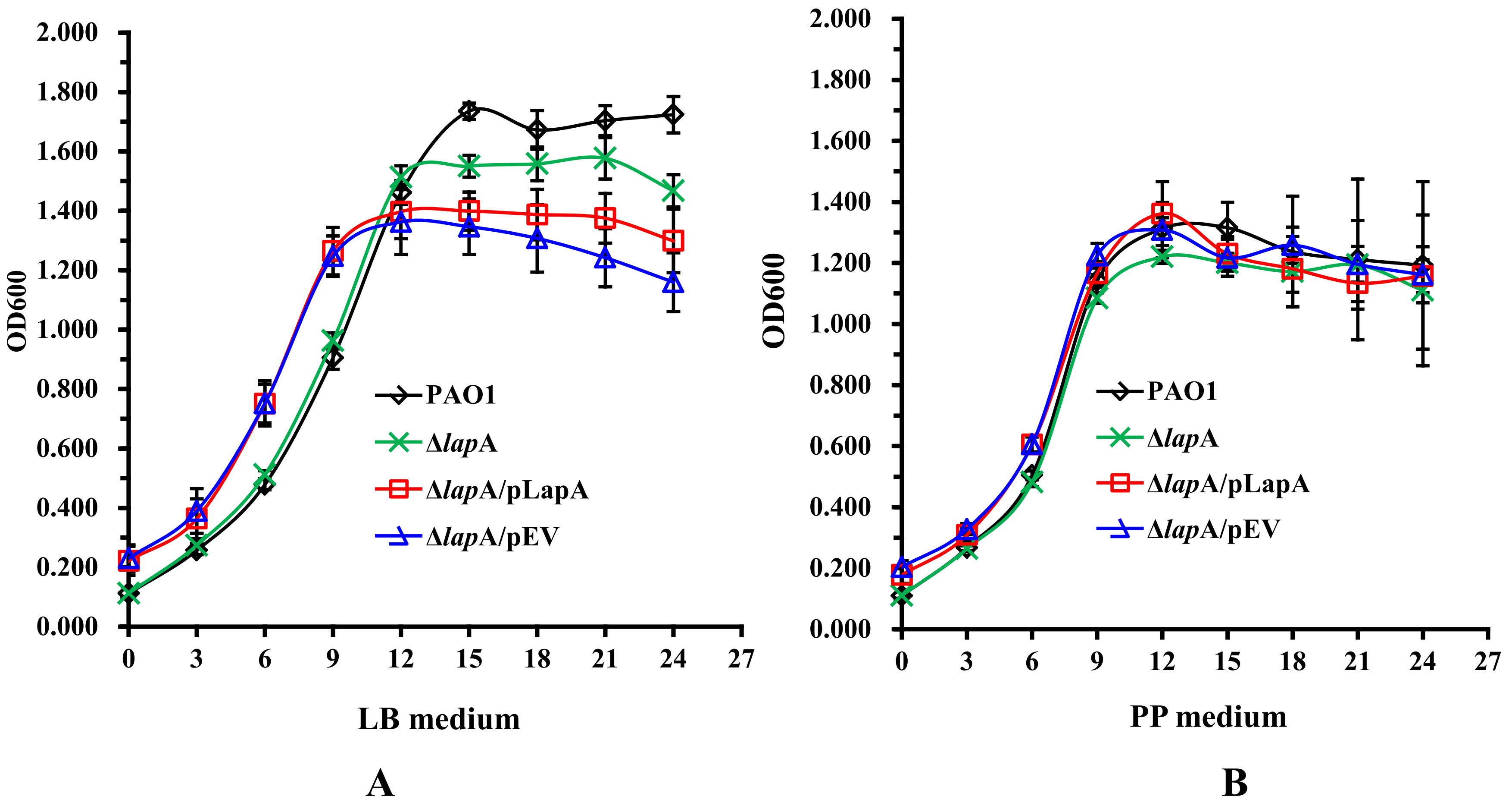

FIG S6 The growth curves of the *Pseudomonas aeruginosa* PAO1, Δ*lap*A, Δ*lap*A/pLapA, and Δ*lap*A/pEV strains incubating phosphate-rich (A) and -depletion (B) media.

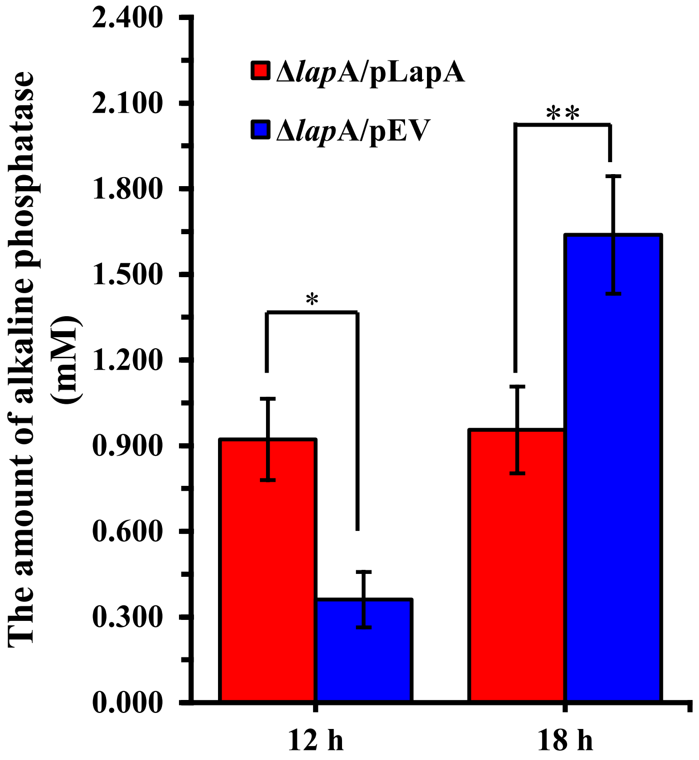

FIG S7 Alkaline phosphatase levels measured in the supernatants from Δ*lap*A/pLapA and Δ*lap*A/pEV strains under phosphate-depleted conditions. The amount of alkaline phosphatase was defined as μmoles of *p*-nitrophenol liberated from *p*-nitrophenyl phosphate at a specific time point. Data are shown as mean ±standard error of the mean of at least three independent experiments. *, $p<0.05$; **, $p<0.01$; ***, $p<0.001$.

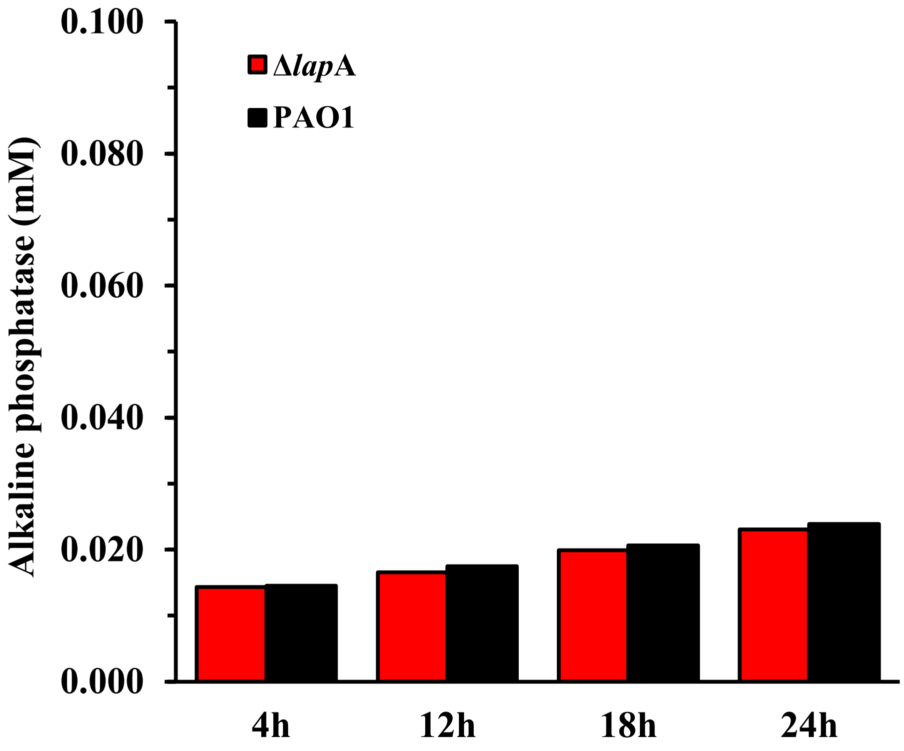

FIG S8 Alkaline phosphatase levels measured in the supernatants from *Pseudomonas aeruginosa* PAO1 and Δ*lap*A strains under phosphate-rich conditions. The amount of alkaline phosphatase was defined as μmoles of *p*-nitrophenol liberated from *p*-nitrophenyl phosphate at a specific time point. Data are shown as mean ±standard error of the mean of at least three independent experiments. *, $p<0.05$; **, $p<0.01$; ***, $p<0.001$.

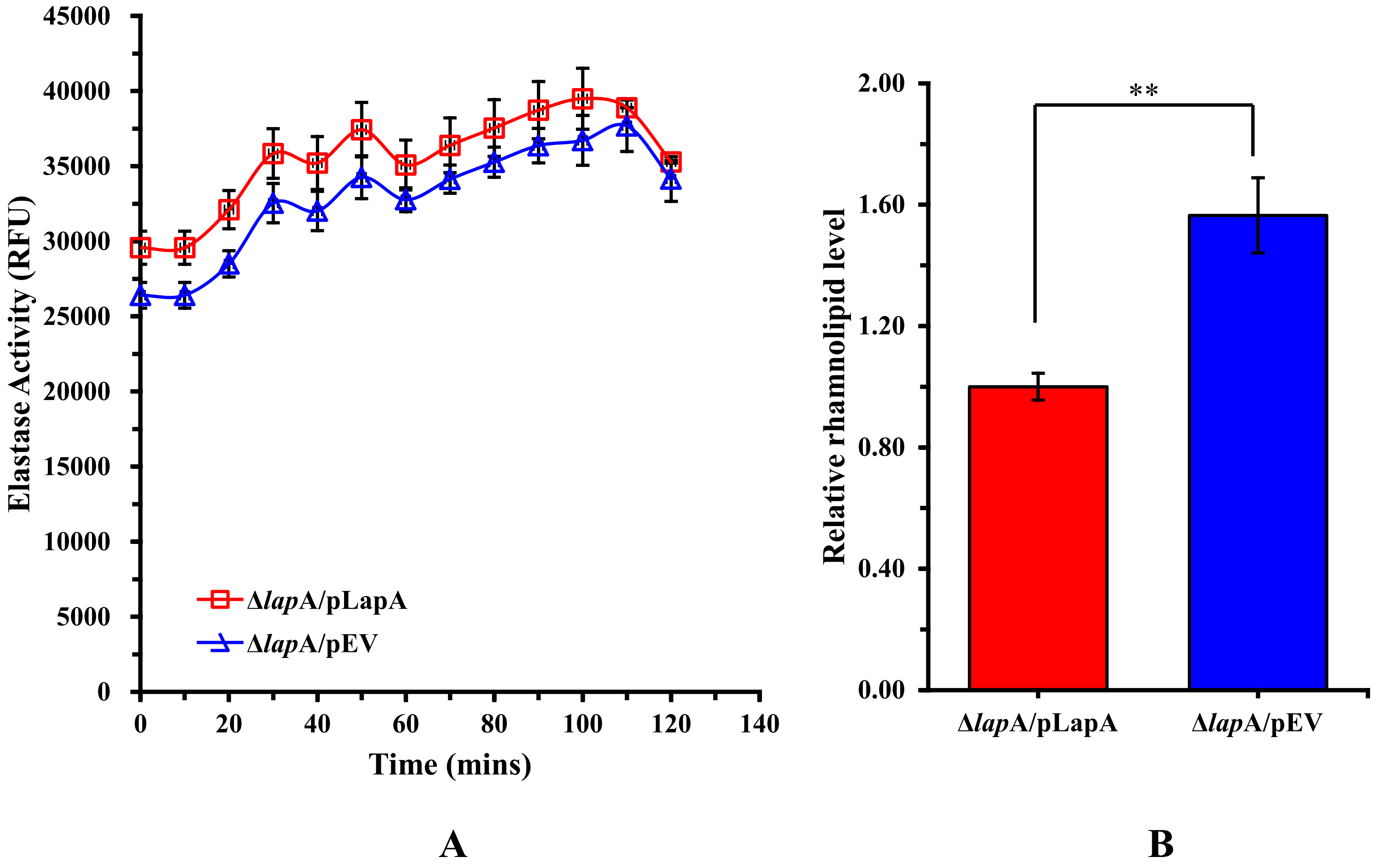

FIG S9 *lap*A gene complementation slightly increased elastase activity (A) and reduced rhamnolipid production of *Pseudomonas aeruginosa* PAO1 under phosphate depletion conditions. Elastase in the supernatants of Δ*lap*A/pLapA and Δ*lap*A/pEV strains cultured under phosphate-depleted conditions for 18 h. Δ*lap*A/pLapA and Δ*lap*A/pEV strains were cultured in phosphate-depleted media for 12 h, and rhamnolipid in the supernatants was determined. Data are shown as mean ±standard error of the mean of at least three independent experiments. *, p<0.05; **, p<0.01; ***, p<0.001.

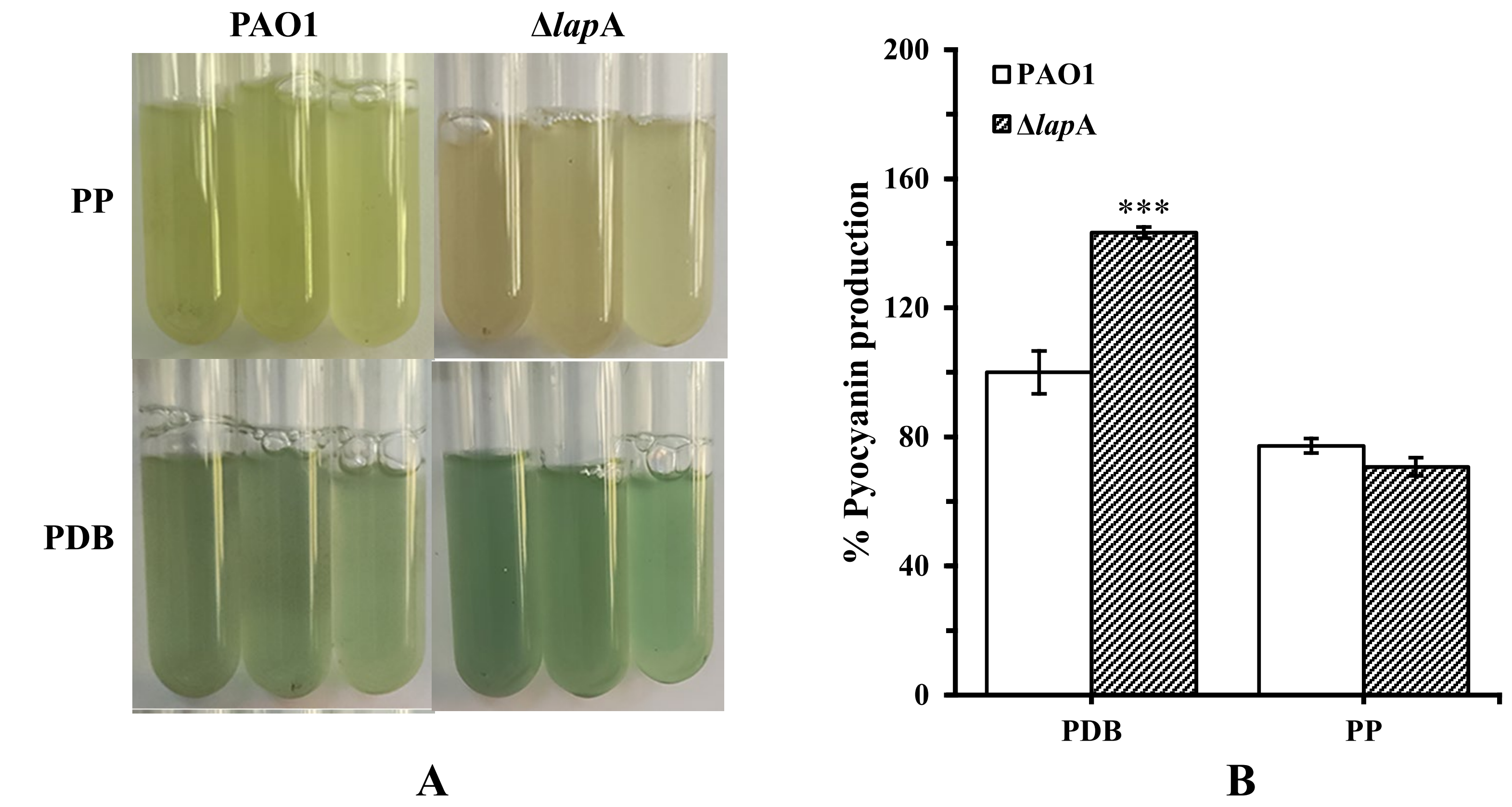

FIG S10 Deletion of *lap*A gene significantly enhanced pyocyanin production of *Pseudomonas aeruginosa* PAO1 when cultured in PDB medium. (A) The colors of WT and Δ*lap*A cultures when incubated in PP and PDB media for 18 h. (B) Pyocyanin production (OD_{695}) was measured in WT and Δ*lap*A strains when cultured in PP and PDB media for 18 h. Data are shown as mean ±SEM of at least three independent experiments. *, p<0.05; **, p<0.01; ***, p<0.001.

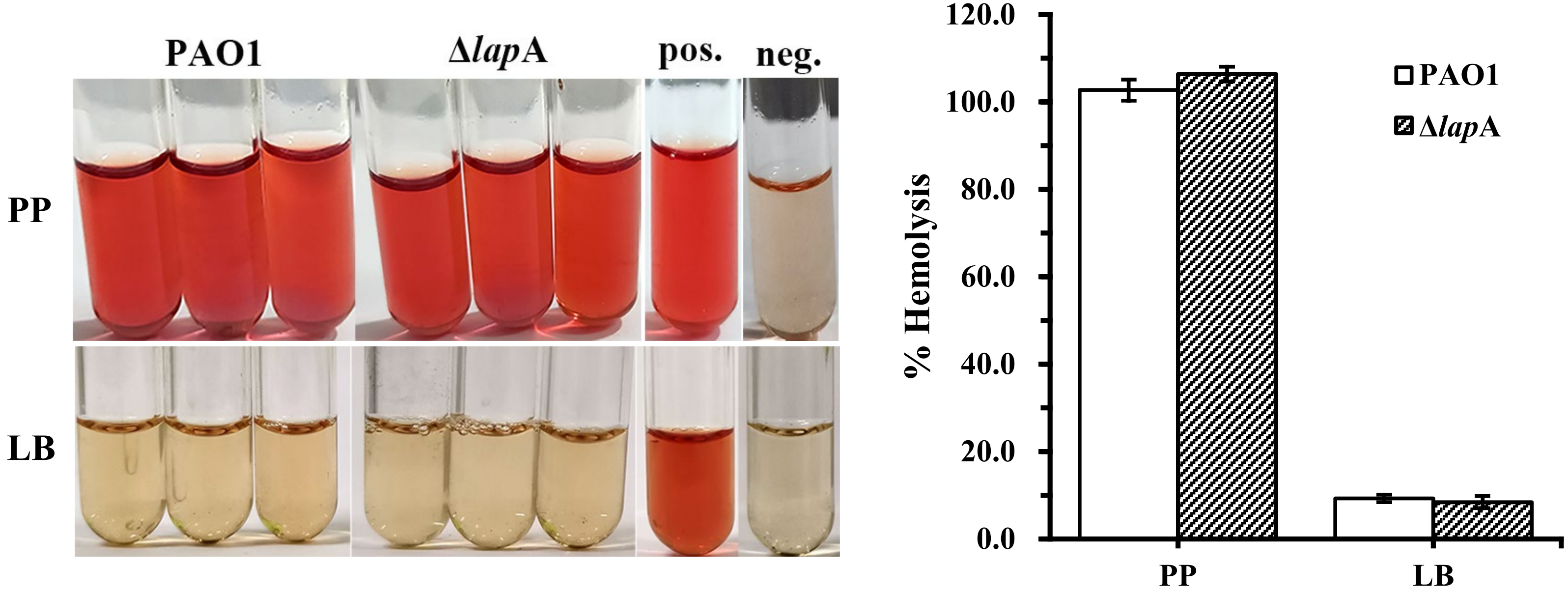

FIG S11 Deletion of *lap*A gene had no effect on hemolysis of *Pseudomonas aeruginosa* PAO1. Hemolytic activity of WT and Δ*lap*A strains that cultured in phosphate-depleted and -rich media for 18 h was detected with 4% sheep blood, 2% TritonX-100 was used as a positive control, and PBS as a negative control. Data are shown as mean ±SEM of at least three independent experiments. *, p<0.05; **, p<0.01; ***, p<0.001.

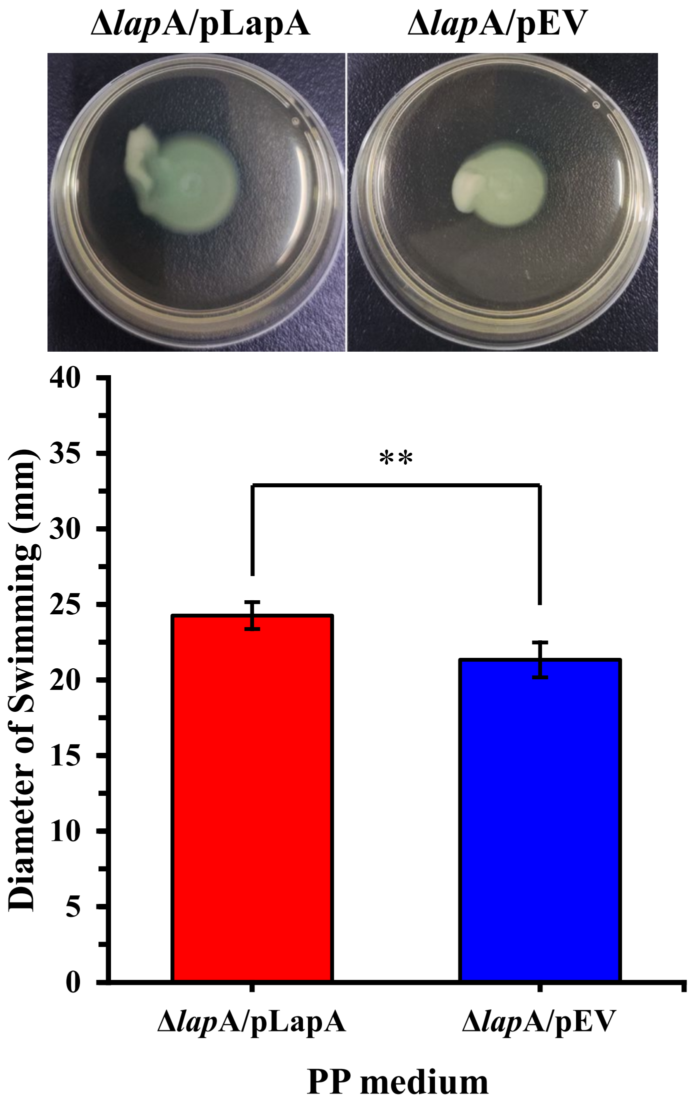

FIG S12 Complementation of the *lap*A gene increased swimming motility of *Pseudomonas aeruginosa* PAO1 under phosphate-depleted stress. One μL of culture was spotted onto the swimming medium without phosphate and incubated for 24 h. Swimming motility was evaluated and the diameter of the halo was measured. Data are shown as mean ±standard error of the mean of at least five independent experiments. *, $p<0.05$; **, $p<0.01$; ***, $p<0.001$.

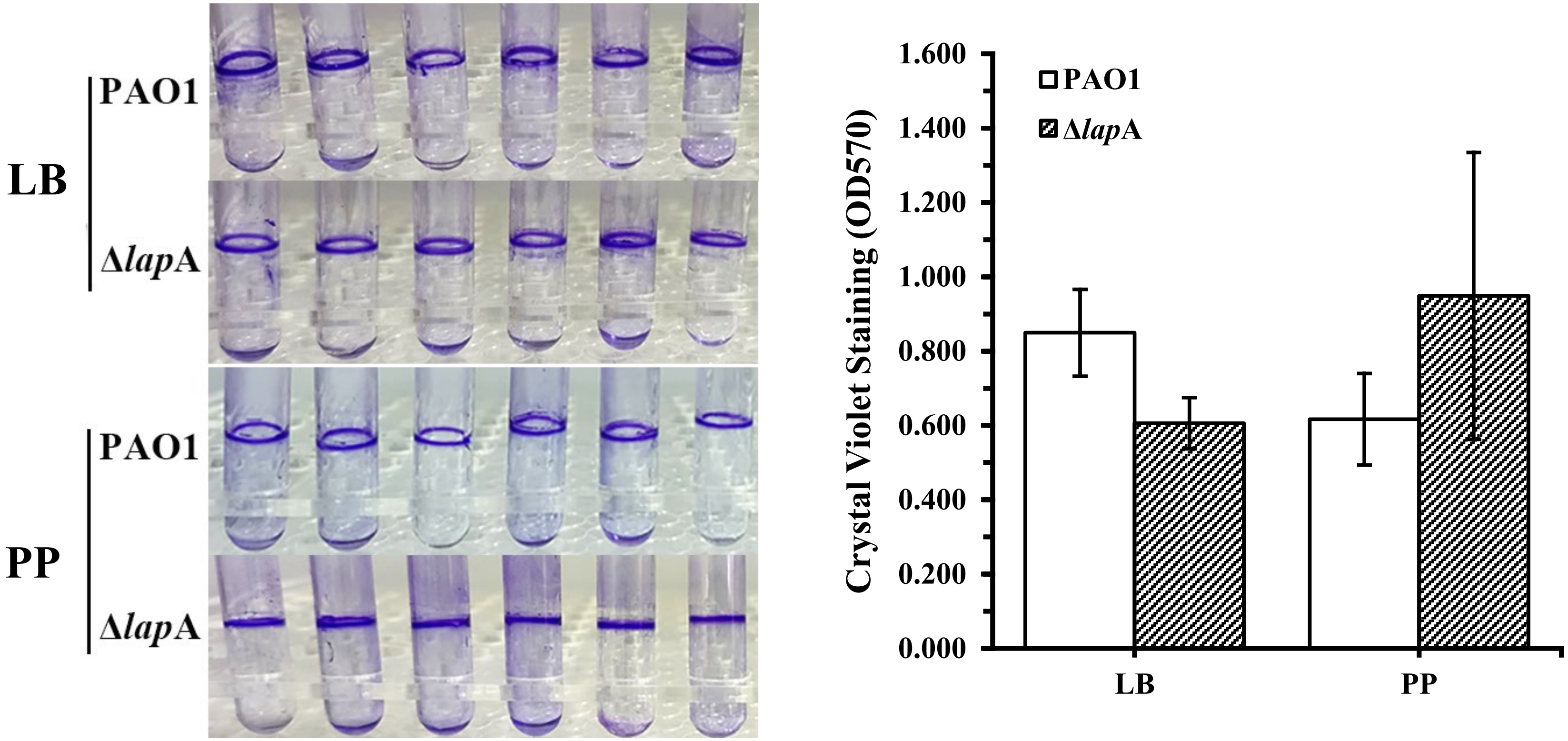

FIG S13 Biofilm formation of Δ*lap*A and WT strains under phosphate-depleted and –rich conditions was displayed with crystal violet staining (left) and quantified with optical density measurement (right). Data are shown as mean ±standard error of the mean of at least five independent experiments. *, $p<0.05$; **, $p<0.01$; ***, $p<0.001$.
