# Supplementary material for: Alkaline phosphatase LapA regulates quorum sensing–mediated virulence and biofilm formation in Pseudomonas aeruginosa PAO1 under phosphate depletion stress
Source: Microbiol Spectr. 2023 Oct 5;11(6):e02060-23. doi: 10.1128/spectrum.02060-23 (PMC10715133; doi:10.1128/spectrum.02060-23)

C4-HSL standard:

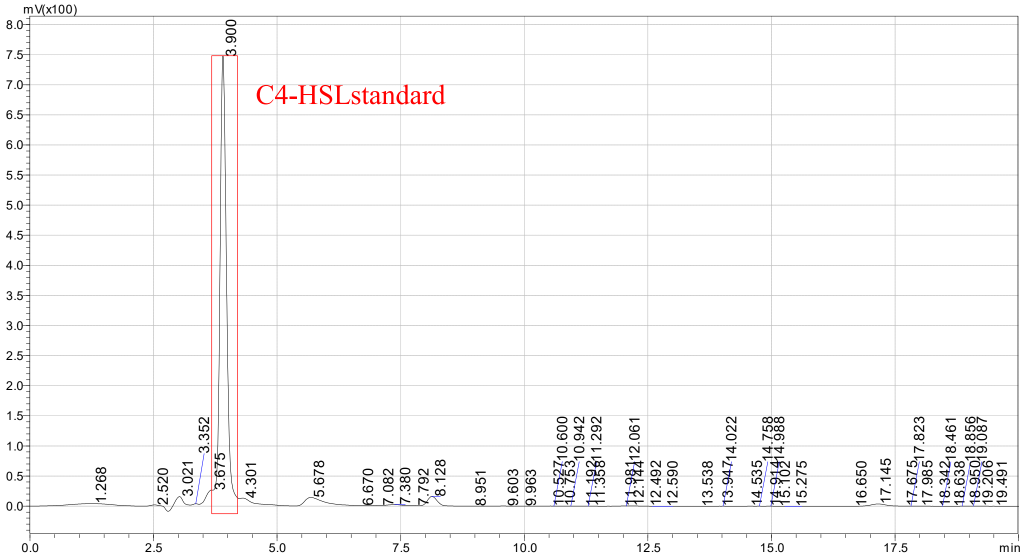

3-oxo-C12-HSL standard:

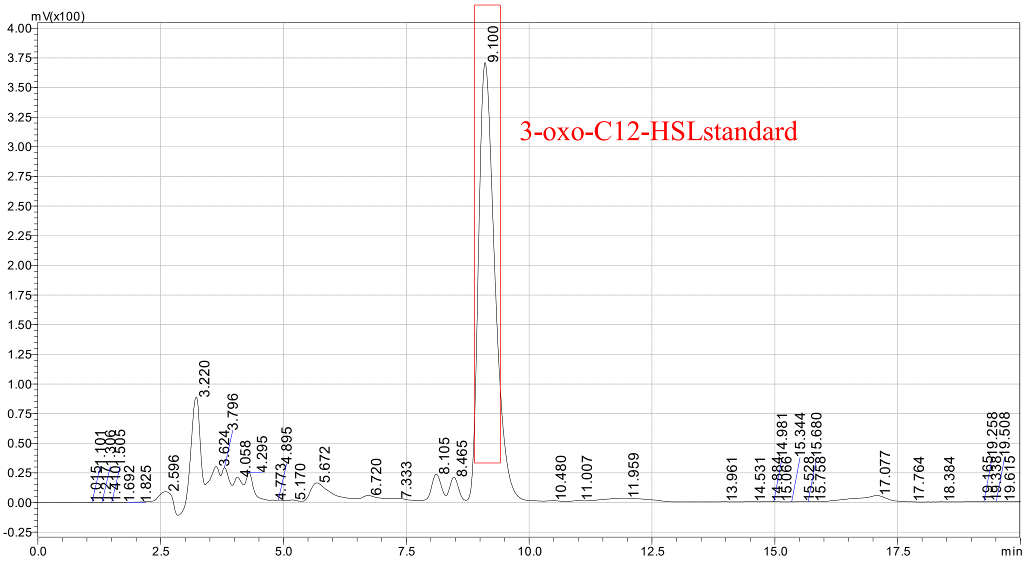

PAO1_LB:

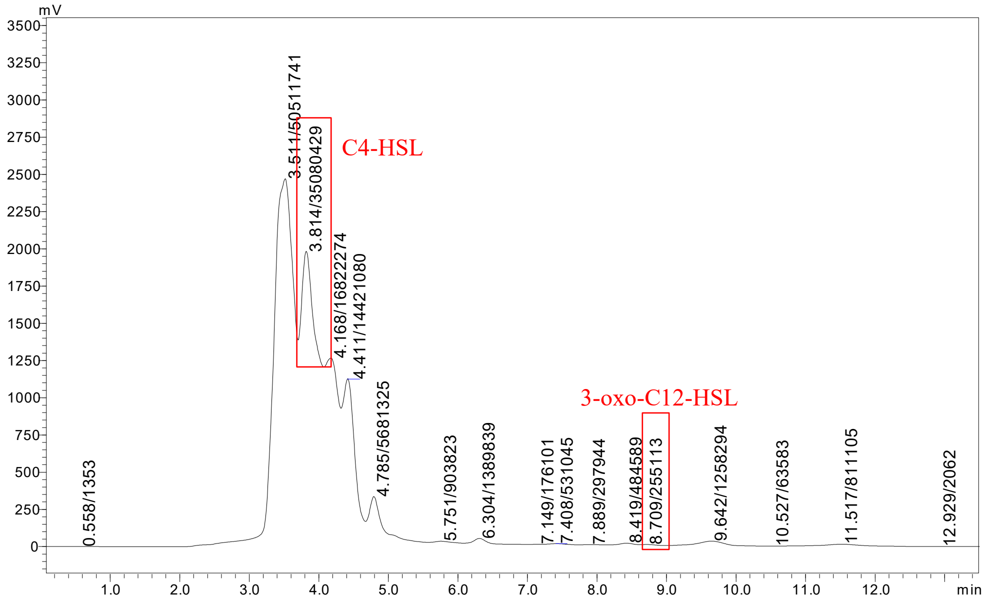

PAO1_LB:

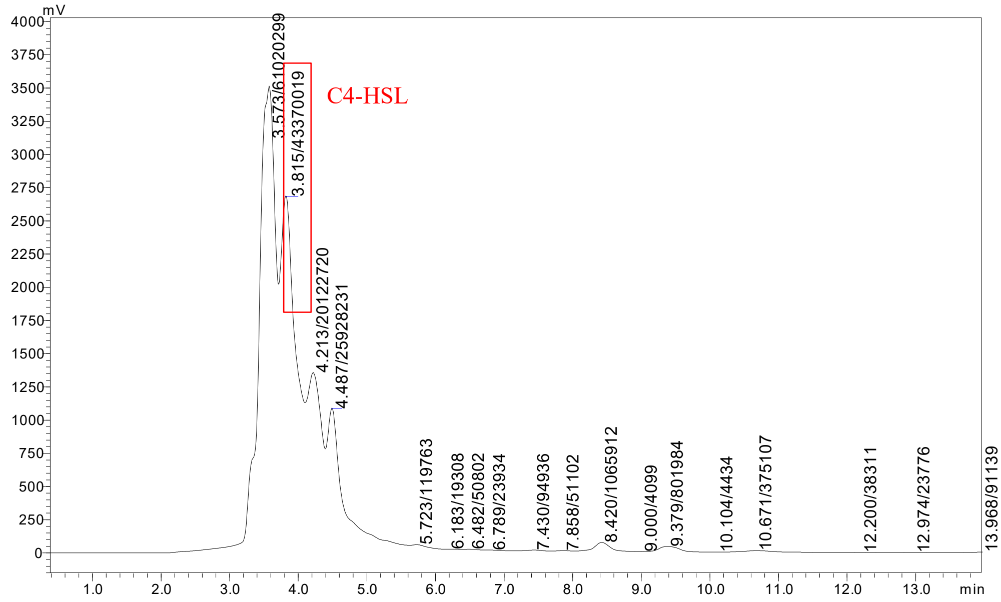

PAO1_LB:

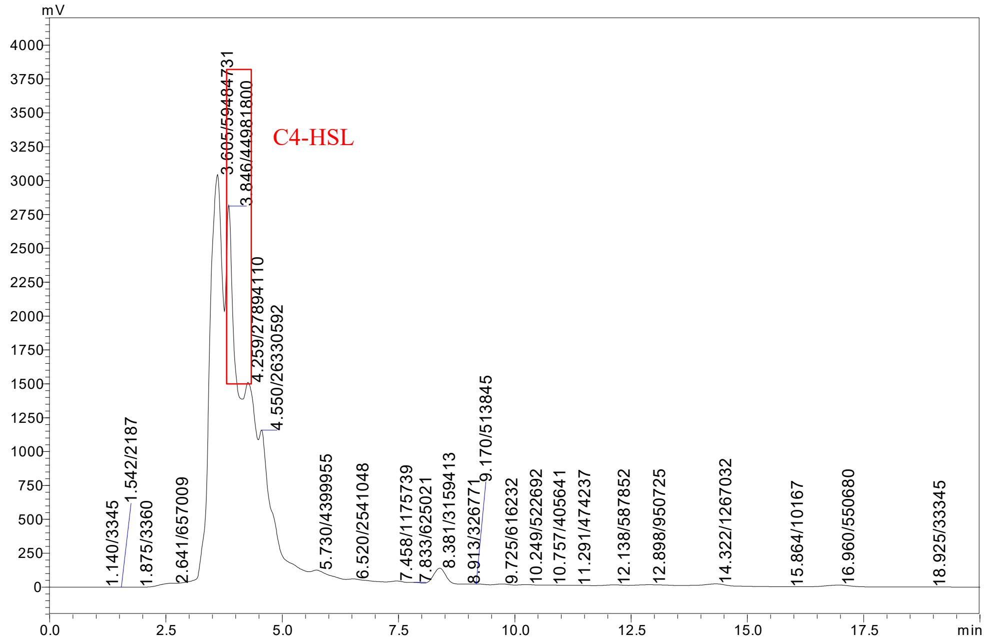

PAO1_LB:

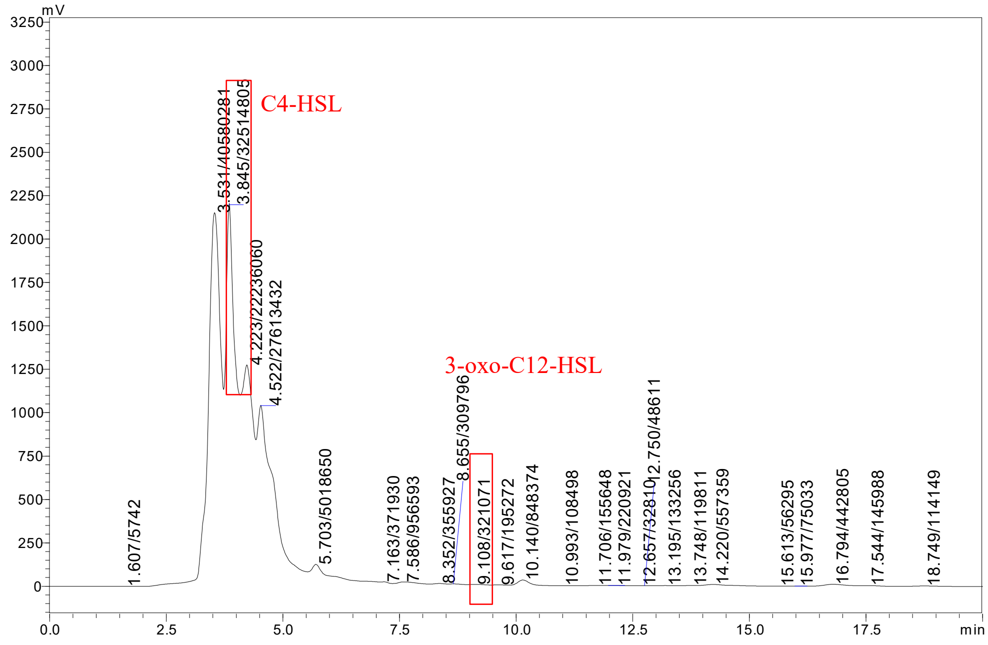

PAO1_PP:

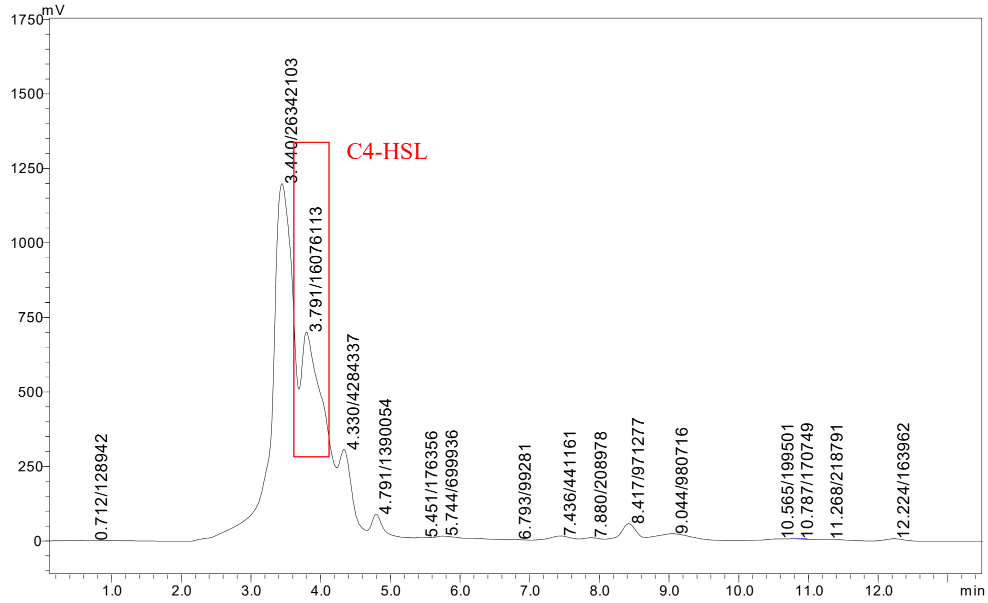

PAO1_PP:

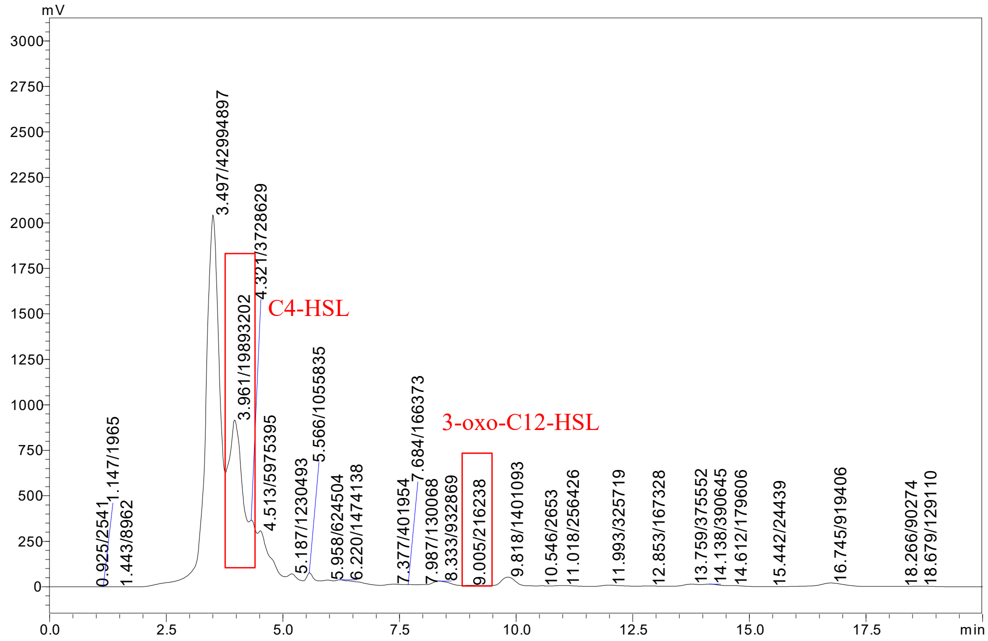

PAO1_PP:

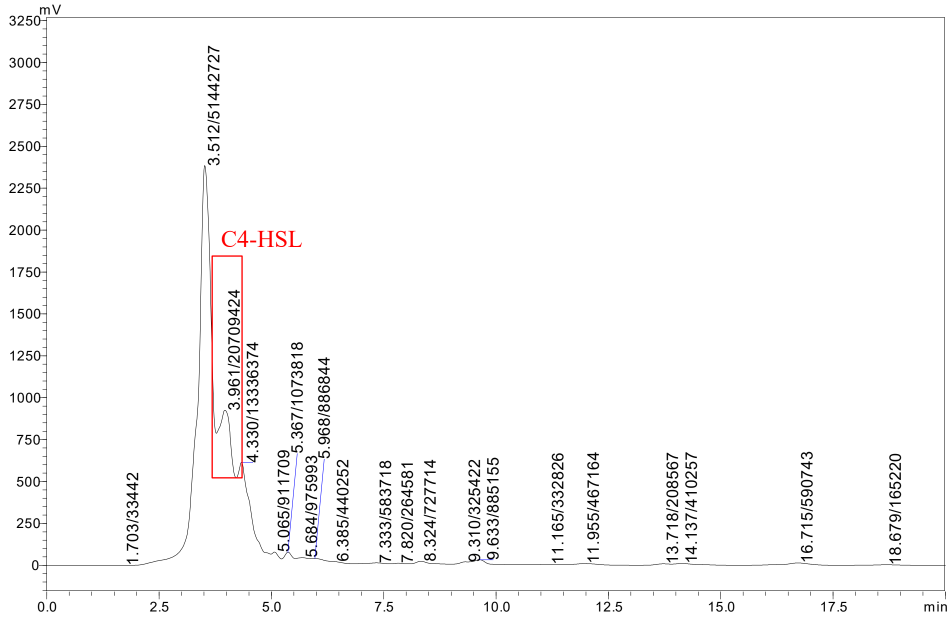

PAO1_PP:

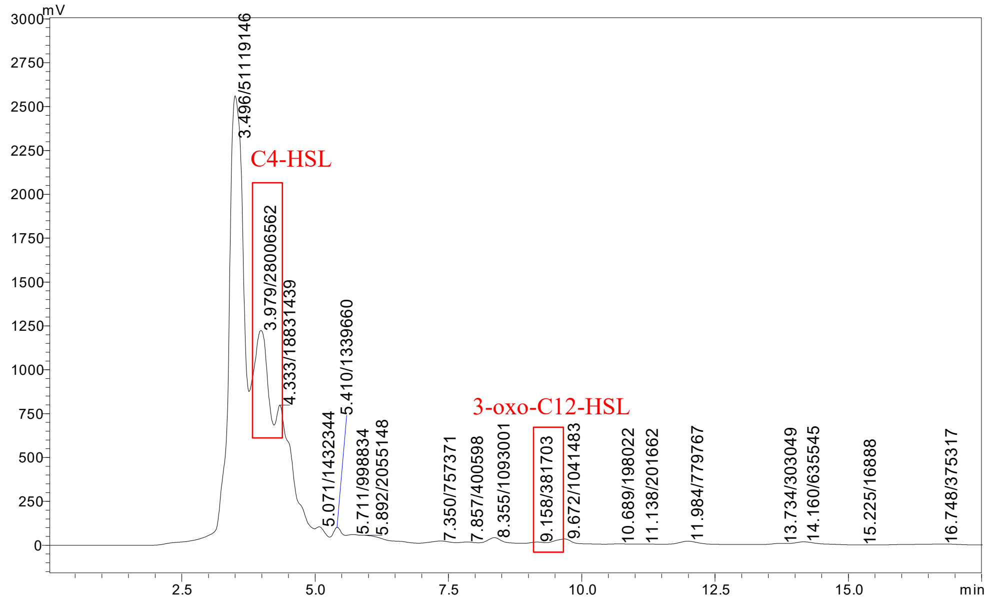

Δ*lap*A_LB:

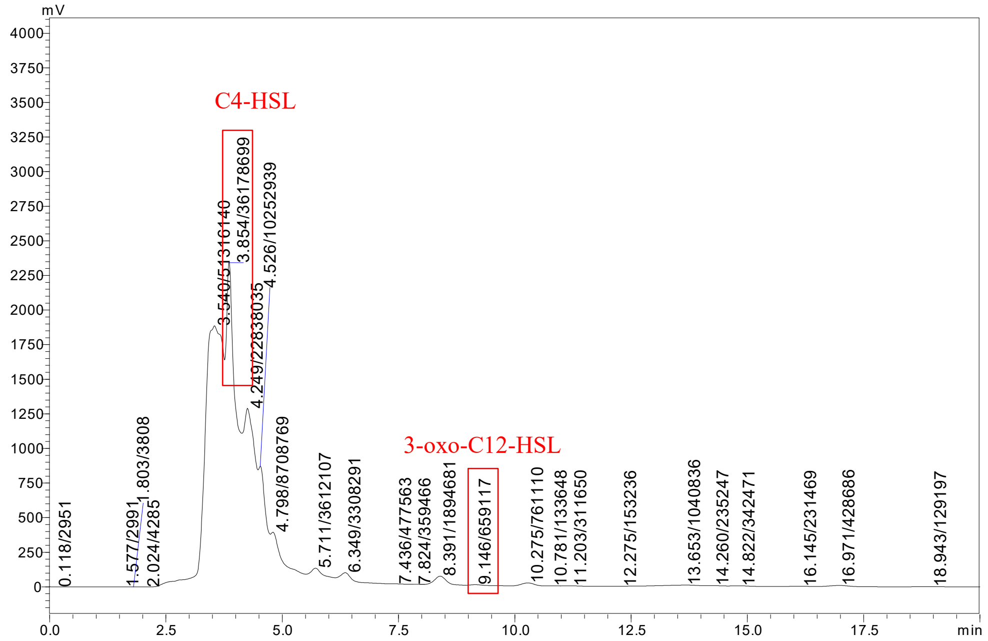

Δ*lap*A_LB:

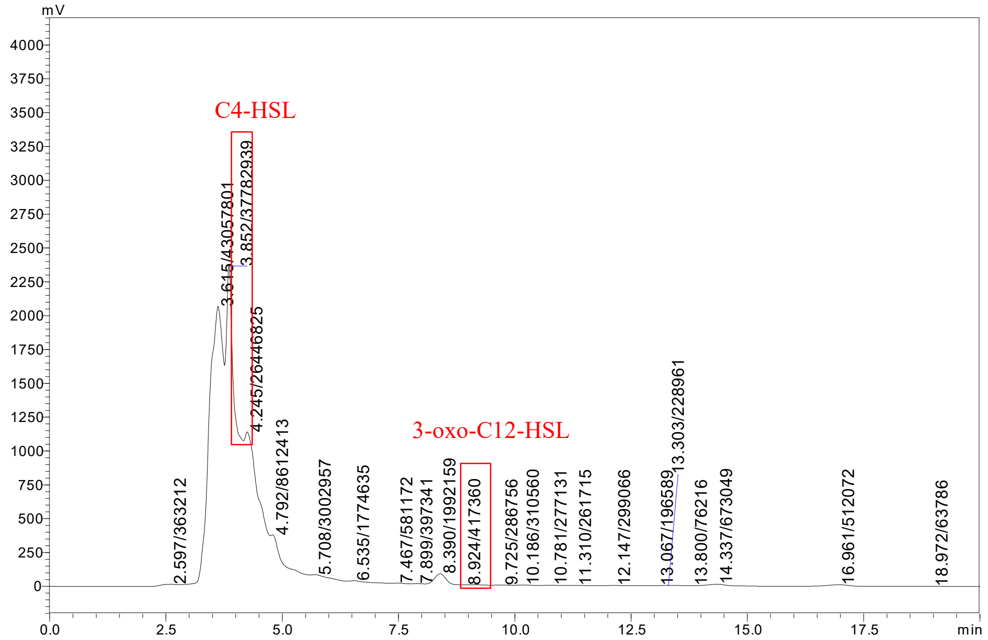

Δ*lap*A_PP:

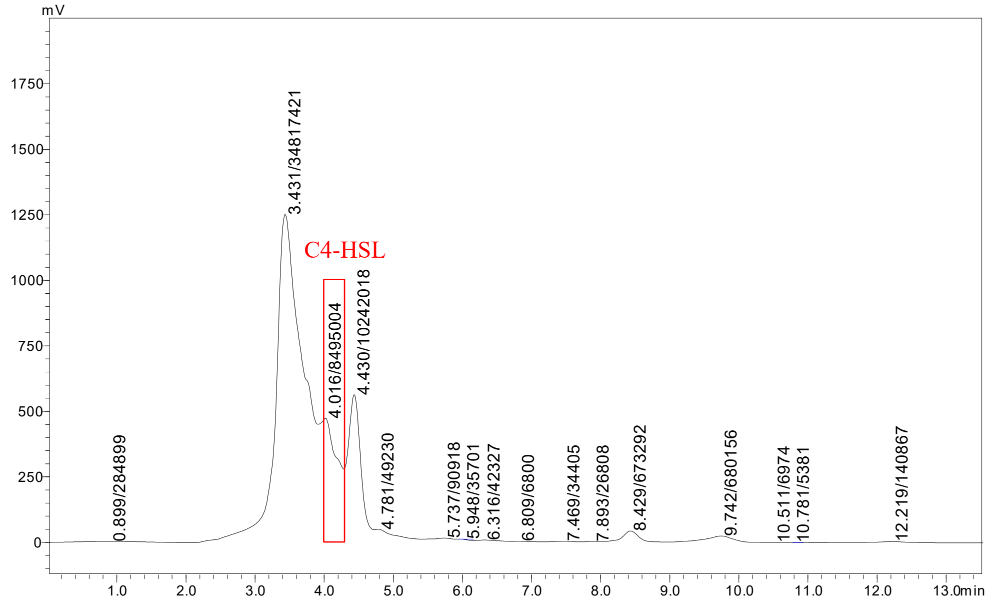

Δ*lap*A_PP:

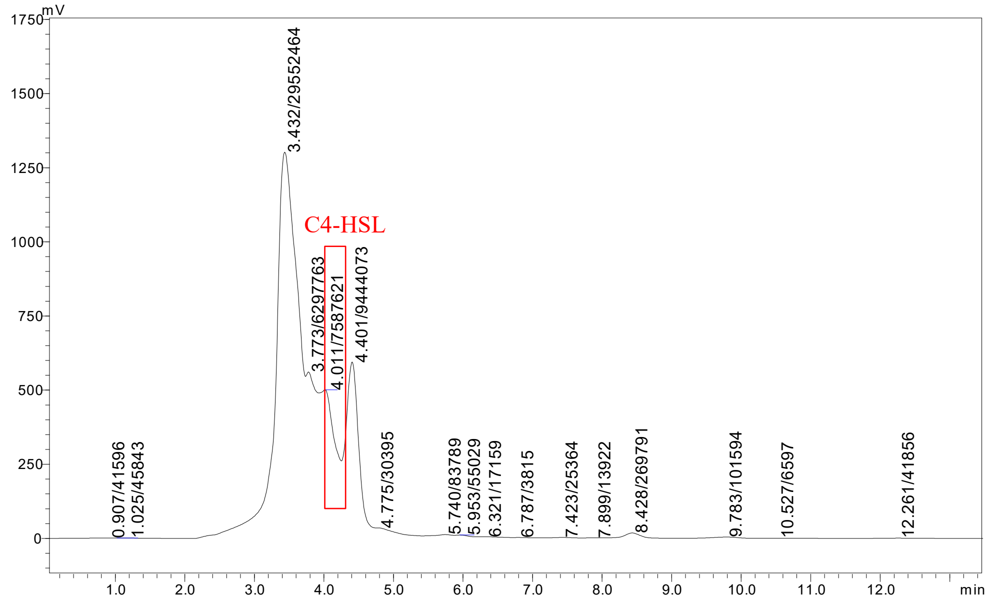

Supplement: Fig. S14 — All HPLC graphs. [file spectrum.02060-23-s0002.pdf]
