# Supplementary material for: Alkaline phosphatase LapA regulates quorum sensing–mediated virulence and biofilm formation in Pseudomonas aeruginosa PAO1 under phosphate depletion stress
Source: Microbiol Spectr. 2023 Oct 5;11(6):e02060-23. doi: 10.1128/spectrum.02060-23 (PMC10715133; doi:10.1128/spectrum.02060-23)
Supplement: Table S1 — Bacterial strains and plasmids used in this study. [file spectrum.02060-23-s0003.docx]

| **Strains and plasmids** | **Genotype or phenotype** | | **Source or reference** |
| --- | --- | --- | --- |
| strains | | | |
| *E. coli* | | | |
| DH5α | | F^–^,φ80,lacZΔM15,Δ(lacZYA-argF)U169,  endA1,recA1,hsdR17(r_k_^–^,m_k_^+^) supE44, λ^–^,thi-1,gyrA96,relA, phoA | Our lab |
| S17-1-*pir* | | RP4-2(Km::Tn7,Tc::Mu-1), pro-82, LAMpir, recA1, endA1, thiE1, hsdR17, creC510 | Our lab |
| OP50 | | Uracil auxotroph, useful for growing *C. elegans* | Our lab |
| *Pseudomonas aeruginosa* | | | |
| PAO1 | | Wild type | Our lab |
| Δ*lap*A | | *lap*A gene deletion strain | This study |
| Δ*lap*A/pLapA | | Δ*lap*A complementation strain | This study |
| Δ*lap*A/pEV | | Δ*lap*A strain containing plasmid pBBR1MCS-5 | This study |
| *Chromobacterium violaceum* | | | |
| CV026 | |  | Our lab |
| Plasmids and vectors | | | |
| pKC1139 | A suicide vector system using the homologous recombination, Apr^R^ | | Our lab |
| pXT01 | Homologous recombination plasmid based on pKC1139, Tc^R^, Apr^R^ | | This study |
| pBBR1MCS-5 | Gm^R^; broad-host-range vector, P_lac_ | | Our lab |
| pLapA | Gm^R^; HidIII/XbaI fragment containing *lap*A *gene* in pBBR1MCS-5 | | This study |
| pCasPA | Tc^R^; used for template for amplifying tetracycline resistance gene | | Our lab |

**Table S1** Bacterial strains and plasmids used in the present study.
