# Supplementary material for: Alkaline phosphatase LapA regulates quorum sensing–mediated virulence and biofilm formation in Pseudomonas aeruginosa PAO1 under phosphate depletion stress
Source: Microbiol Spectr. 2023 Oct 5;11(6):e02060-23. doi: 10.1128/spectrum.02060-23 (PMC10715133; doi:10.1128/spectrum.02060-23)
Supplement: Table S2 — Sequences of the primers used in this study. [file spectrum.02060-23-s0004.docx]

**Table S2** Sequence of the primers used in this study.

| **Primer** | **Sequence (5’-3’)** |
| --- | --- |
| *lap*A_P1 | CCCAAGCTT(HindIII)TCTGCTCGGTCGCTTCGTG |
| *lap*A_P2 | TGCTCTAGA(XbaI)CATGGCTTGGGCGGATACGA |
| *lap*A_P3 | CGCGGATCC(BamHI)ATCGTGAGCGCCTTCTACAC |
| *lap*A_P4 | CCGGAATTC(EcoRI)AGGGAGCAAGCCATTCACTC |
| *lap*A_P5 | TCAACGTACCAACACCACGA |
| *lap*A_P6 | GTGTAGAAGGCGCTCACGAT |
| *lap*A_P7 | CCGGAATTC(EcoRI)TCCGTACCTGAACAAGCCC |
| *lap*A_P8 | CGCGGATCC(BamHI)TTACGGGCGGCCTTTGGTGTT |
| *tet*R-P1 | TGCTCTAGA(XbaI)TCACTGTATTCGGCTGCAACT |
| *tet*R-P2 | CGCGGATCC(BamHI)TATCGTTTCCACGATCAGCGA |
| *las*I-P1 | GCGTGCTCAAGTGTTCAAGG |
| *las*I-P2 | ATTCGCCAGCAACCGAAAAC |
| *las*R-P1 | TCGAACATCCGGTCAGCAAA |
| *las*R-P2 | GTTCACATTGGCTTCCGAGC |
| *las*B-P1 | \| AACCGTGCGTTCTACCTGTT \| \| --- \| |
| *las*B-P2 | GGTCCAGTAGTAGCGGTTGG |
| *rhl*I-P1 | CATCCGCAAACCCGCTACAT |
| *rhl*I-P2 | GGGTTTCGCTGCACAGGTA |
| *rhl*R-P1 | TGAGGAATGACGGAGGCTTT |
| *rhl*R-P2 | AGGCGTAGTAATCGAAGCCC |
| *pls*A-P1 | TGATCTTCTGGTTCACCGGC |
| *pls*A-P2 | GGTACATGCCGCGTTTCATC |
| *pel*C-P1 | TCCAGCTTCACCAGCGAAAG |
| *pel*C-P2 | GCGCCTGGGAATAATTGAGC |
| *psq*R-P1 | ATAGCCTGGCGACGATCAAG |
| *psq*R-P2 | CACTGGTTGAAGCGGGAGAT |
| *rps*L-P1 | TATACACCACCACGCCGAAA |
| *rps*L-P2 | TCACCACCGATGTACGAGGA |
| *rec*A_P1 | GCCAACTGCCTGGTCATCTT |
| *rec*A_P2 | GGCGTAGAACTTCAGTGCGT |
